# Architecture and conformational dynamics of the BAM-SurA holo insertase complex

**DOI:** 10.1126/sciadv.ads6094

**Published:** 2025-04-04

**Authors:** Philippe A. Lehner, Morris Degen, Roman P. Jakob, Seyed Majed Modaresi, Morgane Callon, Björn M. Burmann, Timm Maier, Sebastian Hiller

**Affiliations:** ^1^ Biozentrum, University of Basel, Basel, Switzerland.; ^2^ Swiss Nanoscience Institute, University of Basel, Basel, Switzerland.

## Abstract

The proper folding of outer membrane proteins in Gram-negative bacteria relies on their delivery to the β-barrel assembly machinery (BAM) complex. The mechanism by which survival protein A (SurA), the major periplasmic chaperone, facilitates this process is not well understood. We determine the structure of the holo insertase complex, where SurA binds BAM for substrate delivery. High-resolution cryo–electron microscopy structures of four different states and a three-dimensional variability analysis show that the holo insertase complex has a large motional spectrum. SurA bound to BAM can undergo a large swinging motion between two states. This motion is uncoupled from the conformational flexibility of the BamA barrel, which can open and close without affecting SurA binding. Notably, we observed conformational coupling of the SurA swing state and the carboxyl-terminal helix grip domain of BamC. Substrate delivery by SurA to BAM appears to follow a concerted motion that encodes a gated delivery pathway through the BAM accessory proteins to the membrane entry site.

## Teaser

The holo insertase complex formed by chaperone SurA and the β-barrel assembly machinery has large conformational plasticity.

## INTRODUCTION

Gram-negative bacteria feature a double membrane as a distinguishing architecture (*1*–*5*). The outer membrane (OM) thereby acts as a selective barrier to the environment, allowing passage of small molecules such as nutrients, ions, and signaling molecules but preventing the entry of larger molecules such as proteins or nucleic acids (*6*–*8*). The OM is composed of an asymmetric lipid bilayer with lipopolysaccharide as the main component and integral OM proteins (OMPs) embedded therein. Freshly synthesized OMPs are produced in the cytoplasm by ribosomes, and from there, they are transported through the inner membrane into the periplasm by molecular chaperones and the translocase of the secretion (Sec) pathway (*7*, *9*). After their arrival in the periplasmic space, the OMPs are taken up by periplasmic chaperones SurA and Skp (seventeen kilodalton protein) and subsequently transported across the periplasm to the OM. Notably, the OMPs are kept in an unfolded state during this transport, preventing their aggregation and misfolding (*10*–*14*).

The insertase for folding and insertion of OMPs into the OM is the essential OMP β-barrel assembly machinery (BAM). BAM is a multicomponent complex consisting of five different subunits, the integral membrane protein BamA and the lipoproteins BamB, BamC, BamD, and BamE, which are anchored to the inner leaflet of the OM. BAM recognizes incoming unfolded OMPs by their C-terminal β-signal motifs and folds them through a hybrid barrel mechanism into the membrane (*15*–*24*). BamA has been observed in several different conformations, an “outward-closed” and an “outward-open” state, which differ substantially (*15*, *16*, *18*). In the outward-closed state, strands 1 and 16 are aligned in canonical antiparallel β strand pair conformation, and the lid formed by loop L6 covers the entire pore diameter, such that the BamA lumen is not accessible from the extracellular side. The lateral gate can be open and closed, mediated by a kink of strand 16 at residue G807 (*25*). In the outward-open state, strands 1 and 16 are in contact at the periplasmic edge, but they diverge toward the extracellular side in a V-shape manner, creating a large extracellular opening of the barrel lumen next to the lid loop L6. Previous studies have shown that in the complete BAM complex in detergent micelles, BamA adopts the outward-open conformation (*18*, *26*). The outward-closed state has been observed for the isolated barrel of BamA and for BAM in the presence of the antibiotic darobactin, a compound from the bamabactin family that mimics the β-signal sequence of clients and, thus, blocks the lateral gate region of the BamA barrel (*27*–*29*).

The precise role of these states in client folding is unclear, as the presence of a client further changes the conformations of BamA. On the basis of cross-linked structures where a client is covalently attached to BamA, it seems likely that a client augments to strand 1, forming a hybrid barrel (*21*–*23*, *30*). For the folding of a client to occur, handover of the unfolded and aggregation-prone OMP from SurA into BAM is, thus, crucial. It is, however, not well understood how the chaperone SurA and the large insertase chaperone BAM interact. SurA is a 45-kDa protein composed of an N- and C-terminal core region and two flexible peptidylprolyl isomerase (PPIase) domains (P1 and P2) (*10*, *31*, *32*) (fig. S1). It likely transports its clients in a crevice-like configuration, formed by the three domains. Structural dynamics of SurA in solution are relevant for OMP recognition and have been characterized (*33*), but it remains unclear how this flexibility contributes to client handover. Microscale thermophoresis (MST) analysis showed that SurA interacts with the BAM complex with a dissociation constant (*K*_d_) of 2.6 μM (*34*). Very recently, cryo–electron microscopy (cryo-EM) structures of BAM cross-linked to SurA were determined, informed by computational predictions (*34*–*36*). While these structures gave highly valuable insights into the architecture, they also strongly motivate studies without the use of covalent cross-linking to explore the conformational space of the complex under unbiased conditions.

Here, we demonstrate, using cryo-EM, that SurA can associate with the BAM complex independently of an OMP cargo. We analyze the ensemble of conformational states of the BAM-SurA complex, revealing that SurA docks to BAM via a stable interface on the N-terminal part of its core domain. Despite their interaction, conformational dynamics of both SurA and BAM are preserved. Our data identify conformational coupling between BamC and SurA that emerges in the assembled BAM-SurA holo insertase complex.

## RESULTS

### The chaperone SurA forms a stable assembly with BAM in vitro

**Fig. 1.**
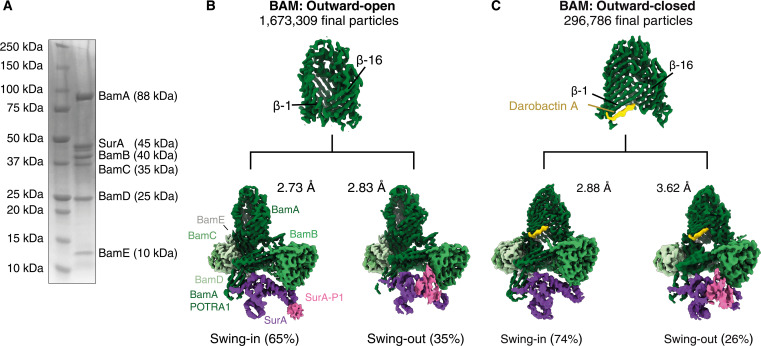
Sample preparation and data processing for the BAM-SurA holo insertase complex. (**A**) SDS-polyacrylamide gel electrophoresis analysis of purified BAM complex mixed with a 1.2× excess of SurA, as used for cryo-EM grid preparation. (**B**) Cryo-EM reconstructions of the BAM-SurA holo insertase complex at representative contour level for every subdomain in absence of darobactin. Under these conditions, the BamA barrel is in the outward-open state. (**C**) Cryo-EM reconstruction of the BAM-SurA holo insertase complex at representative contour level for every subdomain in the presence of darobactin. BAM adopts the outward-closed conformation. BAM subunits are labeled and colored in shades of green, the SurA core domain is purple, the P1 subunit is pink, and darobactin is yellow. Processing details are shown in figs. S2 and S3.

The BAM complex, composed of the five subunits BamA to BamE, was overexpressed in *Escherichia coli*, extracted from the native membrane in detergent micelles and purified to a high degree of purity. We independently expressed and purified the periplasmic chaperone SurA and incubated it with the BAM complex at a 1.2-fold molar excess. The mixture was then directly applied to cryo-EM grids and plunge frozen, and data were collected on a Titan Krios (Fig. 1A). We collected a large dataset of 68,464 movies with 1,673,309 final particles. In this dataset, we observed a holo complex comprising the full BAM complex in detergent micelles with a single-bound molecule of SurA, and the large particle number allowed us to resolve individual conformational states at high resolution.

We used iterative rounds of three-dimensional (3D) classifications at low resolution (~10 to 20 Å) to achieve a clear separation of BAM-SurA particles in distinct conformational states. The resulting classes were further subjected to heterogeneous refinements and lastly underwent nonuniform refinement at full resolution (figs. S2 and S3). This process enabled us to resolve two distinct conformational states of the BAM-SurA complex, which we termed “swing-in” and “swing-out” state, at global resolutions of 2.73 and 2.83 Å, respectively (Fig. 1B, fig. S2, and table S1). In a 3D variability analysis, the transition between these states is represented as a single variability component depicting a swinging motion of bound SurA.

To investigate how these SurA conformations are linked to conformational rearrangements of the BamA barrel, we prepared BAM and incubated it with a 1.5-fold molar excess of darobactin to force the complex into the outward-closed conformation. After subsequent incubation with 1.2-fold excess of SurA and data collection, we applied the same processing strategy as outlined above. As expected, the BAM complex was in the outward-closed state. In addition, this sample showed the same distinct conformational states, a swing-in and a swing-out state, resolved at global resolutions of 2.88 and 3.62 Å, respectively (Fig. 1C and fig. S4). In both samples, the population of the swing-in state with 65 and 74% for outward-open and outward-closed, respectively, was higher than that of the swing-out state (35 and 26%).

While the local resolution for the BAM complex and most of its components (A, B, D, and E) were generally very consistent, the local resolution for SurA core domain and especially the P1 subunit varied depending on the conformational state (fig. S5). In the following, we analyze these four states, i.e., BAM-SurA swing-in, BAM-SurA swing-out, BAM-SurA-darobactin swing-in, and BAM-SurA-darobactin swing-out (Fig. 1, B and C), to describe the conformational space of the BAM-SurA holo insertase complex.

### SurA binds the BAM complex via β strand augmentation

We start our characterization with the swing-in state of the BAM-SurA holo complex. The resolution achieved for this state allowed us to build an accurate molecular model for the BAM complex components A, B, D, and E, as well as for the N-terminal part of BamC and the core domain of the bound SurA chaperone (figs. S5A and S6, A and B). The resolution for the SurA P1 domain (residues 171 to 273) was insufficient for de novo atomic model building. However, its location was well defined in the cryo-EM map to a local resolution of >7 Å, which enabled rigid body fitting with the known structure of the P1 domain. P2 (residues 274 to 389) showed only minimal density in the map, and we concluded that it is flexible, in a multistate equilibrium that cannot further be resolved with the available data (fig. S6C). Notably, the C-terminal helix grip domain of BamC showed considerable density with a local resolution of about 3 to 5 Å (fig. S5C). Several side chains and secondary structure elements were clearly recognizable, such as α helix 1 or β sheet 4 to 6, allowing for rigid body fitting of this domain and atomic modeling for the interface to neighboring domains (fig. S6D).

**Fig. 2.**
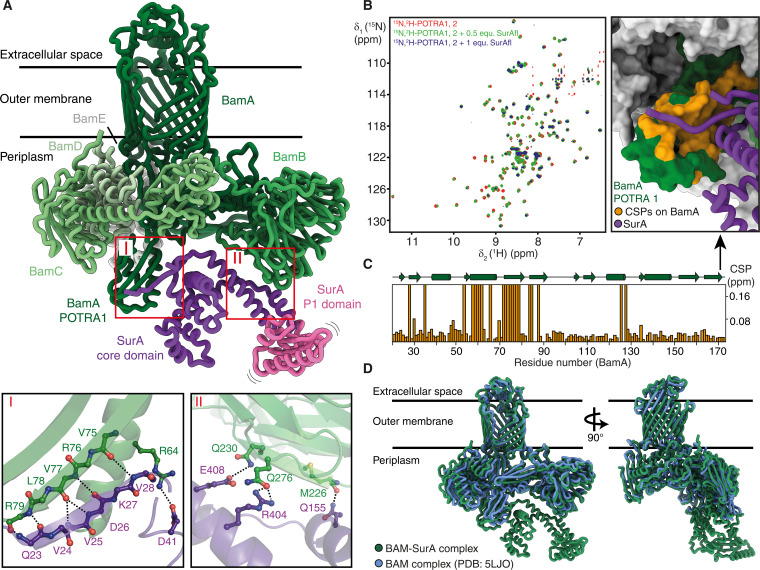
SurA binds BAM to form the holo insertase complex. (**A**) Cryo-EM structure of the BAM-SurA complex in the swing-in state. BAM components, shades of green; SurA core domain, purple; SurA P1 domain, pink. Brackets around the P1 domain indicate the high flexibility of this domain. Key interaction interfaces of SurA and BAM are shown as close-ups (I and II). (**B**) 2D [^15^N,^1^H]-TROSY-HSQC NMR spectrum overlay of 420 μM apo [U-^15^N,^1^H]-POTRA1, 2 in NMR buffer (red), with 0.5 equimolar (equ.) full-length SurA (green) and equimolar full-length SurA (SurAfl; purple). (**C**) Chemical shift perturbations (CSPs; in parts per million) of POTRA1, 2 residues upon titration with SurA, plotted against the POTRA1, 2 residue number. Residues undergoing intermediate exchange upon interaction with SurA were assigned a CSP of 1. Secondary structure elements of POTRA1, 2 are indicated above. Substantial CSPs and peaks undergoing intermediate exchange mapped in orange color on the surface of POTRA1, 2, in the structure from (A). (**D**) Superimposition of the BAM-SurA complex (green) and the apo BAM structure (PDB: 5LJO; blue).

In this swing-in state, SurA and BAM form two key interaction interfaces (Fig. 2A): Interface I is located between the core domain of SurA and the polypeptide-transport-associated domain 1 (POTRA1) and POTRA2 domains of BamA. This interface is formed by full β strand complementation via backbone hydrogen bonding between the β sheet of POTRA1, specifically the β strand V75 to R79 and the N-terminal β strand of SurA (Q23 to V28). An additional prominent interaction includes a salt bridge between R64 of POTRA1 and D41 of SurA. Notably, the relevance of R64 for the BAM-SurA interaction had been identified in early biochemical studies (*37*), and this contact is now fully confirmed with the structure at hand. Our structure further confirms the computational prediction of the POTRA1-SurA interface (*34*, *36*). Interface II is formed between the SurA core domain and BamB. It primarily involves hydrogen bonding between the SurA residues R404, E408, and Q155 with BamB residues Q276, Q230, and M226.

We used solution nuclear magnetic resonance (NMR) spectroscopy to validate interface I by an independent method. A uniformly ^15^N-labeled construct of POTRA1, 2 was titrated with unlabeled SurA and monitored by 2D [^15^N,^1^H]-TROSY-HSQC experiments to detect chemical shift perturbations (CSPs) on POTRA1, 2 upon binding (Fig. 2B). Substantial CSPs were detected in POTRA1, 2 and mapped onto the structure of the BAM-SurA complex. The affected residues correspond well with the position of interface I, where β strand complementation occurs (Fig. 2C), and, thus, confirm this interface. In addition, we probed the role of SurA P2 in the interaction that was not resolved in the cryo-EM structure. We repeated the NMR experiments with a SurA construct with deleted P2 domain and observed essentially the same CSPs, directly proving that the P2 domain neither binds directly nor modulates the interaction (fig. S6E). To characterize the interaction between SurA and the BAM complex, we used surface plasmon resonance (SPR) to determine affinities. The dissociation constant of SurA and POTRA1, 2 was measured to be *K*_d_ = 16 μM (fig. S6F, G). We also observed the interaction of SurA with the BAM complex, but an exact *K*_d_ determination by SPR was not feasible because of nonspecific binding of the chaperone SurA to the SPR chip (fig. S6, H and I). Nevertheless, the data suggest a *K*_d_ in the low micromolar range, consistent with published MST results reporting a *K*_d_ = 2.6 μM (*34*). On the background of our structural data, these affinity measurements show that the interface of SurA to POTRA1, 2 is dominant for the interaction and the interface of SurA with BamB contributes only mildly to the total affinity. The POTRA-SurA interface, thus, forms independently of the rest of the BAM complex and determines the relative orientation of the two proteins.

We next assessed the impact of SurA binding on BAM by aligning our structure to a reference structure of the apo BAM complex structure [Protein Data Bank (PDB): 5LJO]. The comparison revealed that the BAM complex undergoes only marginal changes upon SurA binding, which were not substantial as they were within the resolution differences of the cryo-EM maps (Fig. 2D). We, thus, conclude that SurA binding does not induce substantial structural changes in the BAM complex. Notably, the structural similarity of the SurA core domain and positioning of the P1 domain in the swing-in state and a previously published SurA dimer bound to a peptide substrate (PDB: 2PV3) suggests that this conformation might represent a substrate “accepting” state of SurA, potentially before facilitating its transfer to the BAM complex (*38*). This observation hints at a role for SurA in the early stages of substrate interaction, where it may accept and prepare substrates before insertion into the BAM complex, while other SurA molecules could continue delivering substrates, ensuring a smooth and continuous assembly process (fig. S6J).

### SurA bound to BAM complex can swing to explore its conformational space

Next, we focused on the SurA swing-out state observed with outward-open BAM, which was resolved to 2.83-Å global resolution (fig. S5B). In this state, the BAM complex components A, B, D, and E, as well as the core domain of SurA, are well resolved. The previously discernible density for the C-terminal helix grip domain of BamC was absent, leading to the conclusion that this domain is substantially more flexible than in the swing-in state. In contrast, the P1 domain of SurA was better resolved than for the swing-in state, and we were, thus, able to independently build and refine an atomic model for this domain (fig. S7A, B). The P2 domain of SurA remained unresolved (fig. S7C).

**Fig. 3.**
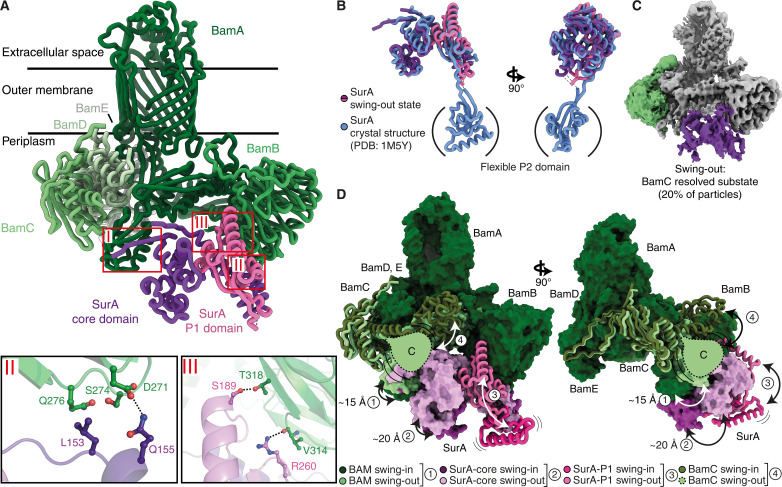
The SurA-POTRA swing motion in the BAM-SurA holo insertase complex. (**A**) Cryo-EM structure of the BAM-SurA complex in the swing-out state. Color code as in Fig. 2. Key interaction interfaces I to III are highlighted. Zoom-ins are shown for interfaces II and III, and interface I resembles to the swing-in confirmation and is therefore not shown. (**B**) Superposition of the SurA swing-out state as part of the BAM-SurA complex (purple) and the SurA crystal structure (PDB: 1M5Y; blue). The bracket indicates that the P2 domain was not resolved in our cryo-EM model due to its inherent flexibility. However, in the SurA crystal structure, this region is resolved. (**C**) Cryo-EM reconstruction of the BAM-SurA swing-out state representing the subset of particles exhibiting partial presence of the BamC subunit. (**D**) Superposition of the swing-in (purple) and swing-out (pink) states of BAM-SurA complex. POTRA1, 2 is shown as dark-green surface in the swing-in state and bright-green surface in the swing-out state. The SurA core domain is depicted as purple surface in the swing-in state and light-purple surface for the swing-out state. SurA-P1 is shown as pink cartoon. BamC is shown as green cartoon—the C-terminal BamC domain for the swing-out state is symbolized by a green shape due to high flexibility. The four substantial structural differences are highlighted by arrows.

The swing-out state differs from the swing-in state mainly in the position of POTRA1, 2 and the SurA core. Furthermore, SurA P1 is located in front of the SurA core next to BamB, and the C-terminal BamC helix grip domain is no longer resolved, potentially because of high flexibility (Fig. 3A and fig. S7D). A further notable difference was found in the interaction triangle BamD, BamC, and POTRA2. In the outward-open conformation of BAM, a salt-bridge forms between D29 of BamD and R162 of BamA. This salt bridge appears to persist in the swing-out state, where the interaction is additionally strengthened by a salt bridge of D29 of BamD with R160 of BamA (fig. S7E). In the swing-in state, this orientation is disrupted and replaced by a salt bridge between D29 of BamD and R271 of the C-terminal helix grip domain of BamC. Meanwhile, the previous interaction partners, R160 and R162 of BamA, have shifted to form hydrogen bonds with Y62 of BamD and S272 of BamC (fig. S7E). This rearrangement suggests a coordinated movement of the C-terminal helix grip domain of BamC, coupled to the swinging motion of BAM-SurA.

Regarding the BAM-SurA interaction, the β strand complementation in interface I is well maintained between the states (fig. S7F), whereas interface II between SurA and BamB undergoes substantial changes. In the swing-out state, the interaction has reduced and comprises only the hydrogen bond between Q155 of SurA and D271 of BamB. However, an additional interface III emerges in the swing-out state between the P1 domain of SurA and BamB including hydrogen bonds of S189 and R260 of P1 and T318 and V314 of BamB (Fig. 3A).

Notably, the arrangement of the SurA domains relative to each other aligns nearly perfectly to an available crystal structure of SurA in the absence of substrate (PDB: 1M5Y) (Fig. 3B) (*32*). This match suggests that the swing-in state could correspond to client-free form of the complex, such as an early stage of the insertion pathway before a client binding or a late stage where the substrate has already been passed onto BAM. This hypothesis is further supported by the absence of clear BamC density in the potential pathway between SurA and BamA in the swing-out state, whereas in the swing-in state, it closely associates with the POTRA2 domain to potentially block a pathway for incoming substrates (Fig. 3C).

The swing-out state differs from the swing-in state in four main ways: First, POTRA1, 2 swings approximately 15 Å away from the center of BAM. Second, a shift of about 20 Å is observed for the SurA core, coupled to the conformational change of POTRA1, 2. Third, the SurA P1 domain moves from its position at the bottom of the complex to adopt a compact structure within a cavity formed by the SurA core and BamB. Fourth, the C-terminal BamC helix grip domain detaches from its well-defined position on POTRA2 into a dynamic ensemble state without coherent density (Fig. 3D, fig. S7G, and movie S1).

### SurA conformational space is independent of the BamA lateral gate state

**Fig. 4.**
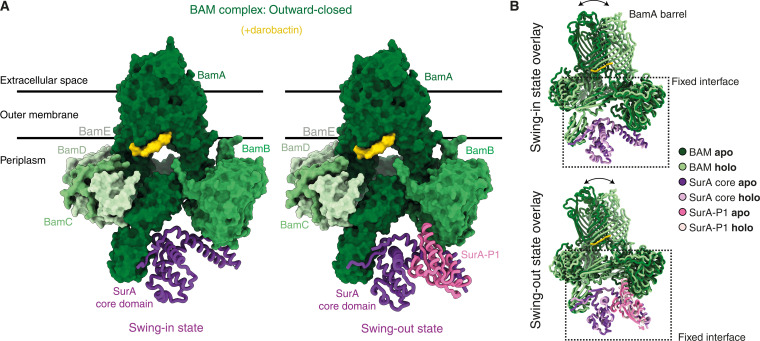
BamA and SurA conformational dynamics are uncoupled in the holo insertase complex. (**A**) Cryo-EM structure of darobactin-bound BAM-SurA complex, with darobactin shown in yellow. Color code as in Figs. 2 and 3. (**B**) Superposition of darobactin-bound and darobactin-unbound state of the BAM-SurA complex for each SurA swing state. A box highlights identical regions between the two structures when aligned to SurA.

Next, we investigated how the conformational space explored by SurA responds to conformational changes of the BamA transmembrane barrel, by analyzing the BAM-SurA complex in the outward-closed state (Fig. 4A and figs. S5, E and F, and S8, A to D). The outward-closed state was induced by incubation with darobactin. This state is an inhibited state that is not necessarily a part of functional insertion pathways but gives experimental access to large-scale rearrangements of the BAM complex. In the outward-closed conformation, we again observed a swing-in and a swing-out state of the BAM-SurA holo insertase complex. In the swing-in state, our refined map achieved a global resolution of 2.88 Å, allowing us to model BamA, BamB, BamD, and BamE, as well as the core domain of SurA. In contrast to the swing-in conformation of the outward-open state, BamC was not resolved in full length, and also P1 and P2 of SurA were not unambiguously recognizable. In the swing-out state density, with a global resolution of 3.62 Å, we were able to model the same components as for the swing-in state with the addition of the P1, while the P2 and BamC domains remain unresolved.

To assess whether SurA binding has an impact on the conformational equilibrium of BAM, we superimposed models for both the swing-in and swing-out states of the BAM-SurA complex in outward-open state with their darobactin-bound counterparts in the closed state (Fig. 4B). The conformational space explored by SurA and the interaction interfaces are fully maintained in both BAM states. The swinging motion of SurA and its interaction with POTRAs and BamB are, thus, fully uncoupled from the conformational rearrangements of the BamA transmembrane barrel (fig. S8, E to G).

## DISCUSSION

**Fig. 5.**
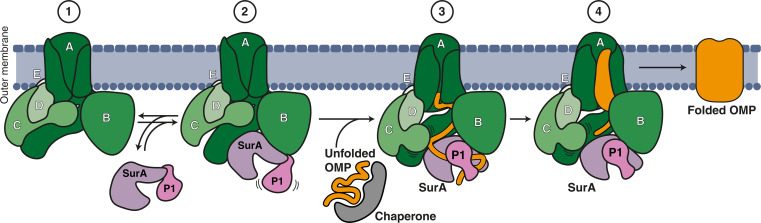
Mechanism of holo insertase–mediated OMP folding and insertion. The proposed mechanism is based on four steps: (1) The apo BAM complex is embedded in the OM. (2) The periplasmic chaperone SurA binds to BAM to form the BAM-SurA holo insertase complex. (3) A second chaperone (e.g., another molecule of SurA, gray) delivers an unfolded OMP substrate (orange) to the complex in the swing-in conformation. The coordinated movement of SurA and BamC to the swing-out conformation facilitates substrate insertion. (4) The OMP substrate is folded and inserted into the OM. SurA remains bound to the BAM complex, awaiting next substrates to arrive.

Overall, our findings suggest that BAM and SurA form a native and stable assembly, the BAM-SurA holo insertase complex, responsible for OMP insertion. This challenges the previous understanding of SurA as being involved primarily in transient interactions with the BAM complex for substrate handover. On the basis of the observation of four distinct structural states and the coupled motion of SurA and BamC in the outward-open state, we propose a mechanism for BAM-SurA holo insertase–assisted folding of OMP substrates (Fig. 5): Initially, in the absence of substrate, SurA binds to the BAM complex. The periplasmic concentration of SurA is approximately 1000 to 3000 molecules per cell, corresponding to a concentration of 20 to 60 μM (*39*). Given the lower copy number of around 800 to 1500 BAM molecules and the affinity of *K*_d_ = ~2 μM, it seems plausible that most BAM complexes are in the holo insertase form at any given time. In the holo insertase complex, SurA can sample both the swing-in and swing-out conformations freely. In the swing-in conformation, the core domain of SurA, along with the P1 and P2 domains, remains exposed and substrate accessible. In this state, BamC interacts with BamD and POTRA2 of BamA (fig. S7E) to effectively block access to the BamA barrel (fig. S9). The substrates are likely transported to the BAM-SurA holo insertase complex by additional copies of SurA or the alternative periplasmic chaperone Skp. Such a client handover mode would align well with the previous findings that SurA can form dimers upon substrate interaction and with the notion that SurA and Skp were found to form transient interactions (*12*, *40*–*42*). These features enable a dynamic handover of the client to SurA in the holo insertase complex. Upon interaction of a substrate, a substantial conformational rearrangement of SurA occurs from the swing-in to the swing-out state of the assembled complex. This transition involves the swing motion of P1 deeper into the core of the SurA binding pocket between POTRA1 and POTRA2 of BamA and BamB subunits. P1 stacks onto the SurA core domain, potentially guiding the associated substrate toward the membrane entry site at the BamA lateral gate. The concomitant motion of BamC may well be necessary for substrate entry by unblocking the passage to the BamA barrel (fig. S9). The coordinated movement of BamC and SurA could, thus, contribute to the productive substrate insertion into the BAM complex, where it can undergo the necessary folding steps to become a fully functional OMP. These rearrangements of BamA and of the folding substrate can readily occur in the swing-out state, as the conformations of the BamA barrel are fully independent of SurA motions. Together with the fact that the OMP folding process is thermodynamically driven, the holo complex may constitute a Brownian ratchet that, using multiple swinging motions of SurA, consecutively guides the substrate toward the lateral gate for subsequent substrate insertion into the membrane. The swinging motion may be essential to maximize the conformational space that the unfolded substrate can explore during this process. After the substrate is successfully folded and inserted, SurA can return to the swing-in conformation, ready to engage with another unfolded substrate. The swing-in state would, thus, correspond to a substrate-expecting state, and the transition to the swing-out state would constitute a Brownian ratchet for client processing. These mechanistic considerations underscore the role of SurA as a critical component of the BAM complex, not merely being a transient “kiss and go”–type mechanism, but an integral part of the holo insertase.

As described above, Radford and colleagues (*35*) recently reported the structure of BAM with covalently cross-linked SurA. They used an AlphaFold2 prediction of the BAM-SurA complex to engineer a disulfide cross-linked complex of BAM with SurA, which they subsequently analyzed by cryo-EM. Multiple structures of differently cross-linked BAM-SurA complexes were resolved at distinct potential stages of OMP folding, including the “wait,” “arrival,” “handover,” and “release” complexes. The wait complexes, captured without any bound substrate, featured an extended and a compact conformation at final resolutions of 4.2 and 4.1 Å, respectively, that are largely congruent with our swing-in and swing-out conformations, highlighting the same interaction mechanism and conformational changes (fig. S10). On the one hand, the higher resolution of the structures described here allows more detailed structural analysis and shows previously unresolved structural features such as the rearrangement of the BamC domain or insights into the relative position of the SurA P1 domain in both conformations, and on the other hand, it justifies the use of cross-links to fix the N-terminal domain of SurA and POTRA1, as performed by Fenn *et al.* (*35*)*.* This implies on one side the biological relevance of these interfaces and strengthens the potential relevance of the arrival complex that used a SurA-OmpX (outer membrane protein X) fusion to trap an early stage of OMP delivery. Together, the two independent studies, thus, demonstrate the interaction of SurA and BAM to form the BAM-SurA holo insertase complex, highlighting its dynamics and biological relevance for OMP insertion.

Further research should aim to incorporate and explore the interactions of the BAM-SurA complex with an unfolded OMP substrate to validate the proposed mechanistic insights and eventually expand them to a variety of different OMPs, including functional studies such as mutagenesis of key interface residues. Most notably, our approach does not use chemical cross-linking between molecules, which may force molecules into artificial conformations. Our study not only advances the understanding of bacterial OMP assembly but also paves the way for future research aimed at targeting the BAM complex for the development of sophisticated antibiotics, potentially offering advanced strategies to combat antibiotic-resistant Gram-negative bacteria.

## MATERIALS AND METHODS

### Expression and purification of SurA

SurA was expressed with a plasmid vector pET28b encoding SurA without the signal sequence (amino acids 21 to 428) and an N-terminal His_6_-tag, followed by a tobacco etch virus (TEV) protease cleavage site. The plasmid was transformed into *E. coli* BL21(λDE3) Lemo cells by heat shock transformation. Cells were grown in LB medium containing kanamycin (30 mg/ml) at 37°C to an optical density of 600 nm (OD_600_) of 0.6. The expression was induced by adding 1 mM isopropyl-β-d-thiogalactopyranoside (IPTG) at 25°C for 16 hours. Cells were harvested by centrifugation (5000*g* for 20 min at 4°C). The cell pellet was resuspended in 25 ml of lysis buffer [25 mM Hepes (pH 7.5), 150 mM NaCl, and 20 mM imidazole] per 1 liters of culture and supplemented with deoxyribonuclease (DNase; 0.01 mg/ml) and inhibitor cocktail (cOmplete EDTA-free protease inhibitor, Roche). The sample was incubated for 30 min on ice and subsequently lysed using a microfluidizer (Microfluidics) for 3 cycles at 4°C. The soluble lysate was separated from cell debris by centrifugation at 14,000*g* for 30 min at 4°C. The supernatant was passed through a 0.20-μm filter supplemented with 8 M urea and loaded onto a preequilibrated Ni–NTA (nitrilotriacetic acid) column. The column with the sample was washed with 20 column volumes (CV) of lysis buffer and eluted with 5 to 10 CV of elution buffer [25 mM Hepes (pH 7.5), 150 mM NaCl, and 500 mM imidazole]. The protein-containing fractions were collected and dialyzed for 16 hours at 4°C in 4 liters of lysis buffer to remove imidazole. Subsequently, the sample was centrifuged at 13,000*g* for 30 min at 4°C. Urea was added to the sample to a final concentration 6 M. The denatured sample was loaded onto a preequilibrated Ni-NTA column and washed and eluted with the same steps as previously but containing 6 M urea in all buffers. The protein containing fractions were again collected and dialyzed for 16 hours at 4°C in 4 liters of lysis buffer to remove imidazole. The sample was incubated for 8 hours at 4°C with 1 mg of TEV per 50 mg of SurA, 0.5 mM EDTA, and 1 mM dithiothreitol to cut of the His_6_-tag. The His-tag was removed by reversed Ni-NTA column, followed by a dialysis with 4 liters of lysis buffer for 16 hours at 4°C. Last, the sample was further purified with a size exclusion chromatography (Superdex 200 16/600 pg) step using 25 mM tris (pH 7.5) and 150 mM NaCl as buffer.

### Purification of the BAM complex

For the expression of the BAM complex, a chemically synthesized (GenScript) single plasmid containing all five *E. coli* BamA to BamE proteins was used, in which BamE was C-terminally linked to a His_6_-tag and BamB C-terminally linked to a Strep-tag II. The BAM complex was expressed in *E. coli* BL21(λDE3) C41 cells in LB medium in the presence of ampicillin (100 μg/ml). Cells were grown at 37°C until an OD_600_ of 0.6, then induced with 0.1 mM IPTG, and further cultivated overnight at 20°C. Cells were resuspended in ice-cold tris-buffered saline [TBS; 50 mM tris (pH 8.0) and 300 mM NaCl], homogenized with a douncer, and lysed using a microfluidizer. In the first centrifugation step, cell debris was removed (7000*g* for 15 min). In the second centrifugation, membranes were pelleted by ultracentrifugation with a 45-Ti rotor (220,000*g* for 2 hours at 4°C). The BAM complex was extracted from the pellet with TBS containing 3% (v/v) ELUGENT (Calbiochem) by shaking for 3 hours at room temperature. After ultracentrifugation with a 45-Ti rotor (100,000*g* for 30 min at 4°C), the supernatant was passed through a 0.22-μm filter, loaded onto a 25-ml Ni Sepharose FF column, washed with 20 CV of TBS containing 0.05% *n*-dodecyl β-d-maltopyranoside (DDM) and eluted with TBS containing 0.05% (w/v) DDM and 400 mM imidazole. The eluate was directly loaded onto a 10 ml Strep-Tactin XT column (IBA) and eluted with 10 mM biotin. Last, the BAM complex protein was purified on a 16/600 Superdex 200 pg column (Cytiva) using 20 mM tris (pH 8.0), 150 mM NaCl, and 0.05% (w/v) DDM, concentrated to 5 mg/ml in Amicon concentrators (Merck), and directly flash frozen in liquid nitrogen.

### EM sample preparation and data collection

Immediately before grid preparation, gold Quantifoil grid 2/1 μm was plasma cleaned using a Solarus Plasma Cleaner 950 (Gatan). For the BAM-SurA sample, a 3.5-μl aliquot of BAM complex at a concentration of ~5.0 mg/ml (~25 μM) was mixed with a 1.2× excess of SurA, applied to the grid (Quantifoil Au200 R2/1) at 16°C and 95% humidity, and blotted and vitrified by plunging into liquid ethane using a Vitrobot (FEI, Vitrobot Mark IV). For BAM-SurA-darobactin sample, the BAM complex was first mixed with a 1.5× excess of darobactin and incubated for 10 min, then a 1.2× excess of SurA was added, and the sample was plunge frozen.

Data were collected on a Thermo Fisher Scientific Titan Krios G4 electron microscope operated at 300 kV, equipped with a Falcon 4i Detector and a Selectris X imaging filter operated with a slit width of 10 eV. Images were acquired at −0.6 to −2.0 μm defocus and a nominal magnification of ×165,000, which corresponds to a pixel size of 0.73 Å. Movies were collected with a total accumulated dose of approximately 40 e^−^/Å^2^, fractionated over 52 frames, using beam-image shift. For BAM-SurA sample, two datasets were collected, and for BAM-SurA-darobactin sample, a single dataset was collected.

### Cryo-EM data processing and model building

For the BAM-SurA sample, two datasets (10,000 and 58,464 movies) were collected, both corrected for beam-induced drift using Patch Motion, and the contrast transfer function (CTF) parameters for each micrograph were determined using Patch CTF in cryoSPARC (*43*). We first processed the smaller 10,000-micrograph dataset. Particles were first picked with the blob picker function in cryoSPARC and subjected to reference-free 2D classification. 2D classes showing structural features were used as templates for particle picking using the template picker in cryoSPARC. After iterative 2D classification, an ab initio reconstruction was used for heterogeneous refinement and 3D classification, separating the swing-in and swing-out conformations. Both conformations were used again for a second round of template picking, followed by 2D and 3D classification and heterogeneous refinement, revealing two substates with different BamC protein conformations for both the swing-in and swing-out conformations.

For the second larger BAM-SurA dataset (58,464 movies), the micrograph denoiser became available in cryoSPARC and was used before particle picking. The overall workflow was similar to the processing of the first dataset, and 3D classification also revealed in total two BAM complex states, each with two substates for the BamC subunit. Now, the particles from both datasets were combined, totaling 584,508 particles for the swing-out and 1,088,801 particles for the swing-in conformation. Using nonuniform refinement, followed by local refinement and global and local CTF refinement, a final map resolution of 2.83 Å (swing-out) and 2.73 Å (swing-in) was obtained. We used local masking, particle subtraction, and local refinements to improve the local resolution. DeepEMenhancer (*44*) was used to improve map quality for model building. For the BAM-SurA-darobactin data, we followed the same strategy and obtained a final map at 3.62 Å for the swing-out state (75,794 particles) and 2.88 Å for the swing-in state (220,992 particles). For the BAM-SurA-darobactin data, we could only resolve the N-terminal domain and part of the linker helix but not the C-terminal domain of BamC.

Modeling was done by manually fitting the BAM complex domains of 5LJO.pdb or 7NRI.pdb (*28*) for darobactin-BAM into the respective EM density maps in UCSF ChimeraX (*45*). The truncated BamC present in 5LJO was replaced by an AlphaFold prediction (*46*, *47*) of the full-length domain. An AlphaFold prediction of SurA was used for initial fitting of SurA into the respective density.

Model building was performed using ISOLDE (*48*) and Coot (*49*), and real-space refinement was carried out in PHENIX (*50*, *51*). Validation was done using the cryo-EM validation tools within PHENIX (*52*–*54*).

### Expression of SurA and POTRA1, 2 for NMR studies

SurA and POTRA constructs were cloned in the pET28b vector (Novagen), containing a thrombin-cleavable N-terminal His_6_-tag. *E. coli* BL21(λDE3) Lemo cells were transformed and grown at 37°C in medium containing kanamycin (30 μg/ml) to an OD_600_ of approximately 0.6 and then for an additional 30 min at 25°C. Expression was induced by 0.4 mM IPTG. Cells were harvested 18 to 20 hours after induction and resuspended in buffer containing 25 mM Hepes, 300 mM NaCl, and 10 mM imidazole at pH 7.5 at a 4:1 buffer/pellet weight ratio and lysed by two passes through a French press. The lysate was centrifuged at 34,500*g* for 1 hour at 4°C, subsequently applied to a 5 ml Ni^2+^-HisTrap (GE Healthcare) column, and eluted with an imidazole gradient. SurA elutes at 150 mM imidazole concentration. The elution fractions containing SurA were dialyzed overnight against buffer A [25 mM Hepes and 150 mM NaCl (pH 7.5)] at 4°C. Dialyzed SurA was denatured with 6 M guanidinium (Gdm)/HCl, applied to Ni^2+^ beads, and eluted with 200 mM imidazole. The eluted SurA was dialyzed overnight against buffer A [25 mM Hepes and 150 mM NaCl (pH 7.5)] at 4°C. Dialyzed SurA was concentrated in a Vivaspin concentrator (molecular weight cutoff, 30,000; Sartorius) and applied to a HiLoad 16/600 Superdex 75 or 200 pg (GE Healthcare). Afterward, eluted fractions containing SurA were concentrated by ultrafiltration and stored at −80°C until use. Expression and purification of POTRA1, 2 were done as for SurA, with the difference that purification under denaturing conditions was replaced by purification via anion-exchange affinity with a 5-ml Q HiTrap FF (GE Healthcare) column and eluted with a NaCl gradient. The POTRA1, 2 construct eluted in the flow-through. Isotope-labeled [U-^15^N]-POTRA1, 2 construct was obtained by growing the expression cells in M9 minimal medium supplemented with (^15^NH_4_)Cl. All isotopes were purchased from Sigma-Aldrich or Cambridge Isotope Laboratories.

### NMR spectroscopy

NMR spectra were recorded at 37°C on Bruker Ascend II 700 and Bruker Avance 900 spectrometers equipped with cryogenic triple-resonance probes. The 2D [^15^N,^1^H]-TROSY-HSQC were recorded in a total experiment time of 2 hours. The ^1^H carrier was centered on the water resonance and the ^15^N carrier at 118 parts per million (ppm). The interscan delay was set to 1 s. In the direct dimension, 1024 complex points were recorded in an acquisition time of 91 ms, multiplied with a 75° shifted sine bell, zero-filled to 2048 points, and Fourier transformed. In the indirect dimension, 256 complex points were measured with a maximal evolution time of 4.44 ms, multiplied with a 75° shifted sine bell, zero-filled to 256 points, and Fourier transformed. For the SurA-POTRA titration series, 2D [^15^N,^1^H]-TROSY-HSQC spectra were recorded with 0.5 and 1 M equivalents of the titrated protein. CSPs of amide moieties were calculated as

$$\text{CSP}=\sqrt{{\left(\delta H_{\text{ref}}-\delta H\right)}^{2}+{\left(\frac{\delta N_{\text{ref}}-\delta N}{5}\right)}^{2}}$$

### Expression and purification of membrane scaffold proteins

The pET28a plasmid encoding MSP1D1 with N-terminal His-tag and cleavage site was transformed into *E. coli* BL21(DE3) cells. An overnight culture was induced with 0.5 mM IPTG at an OD_600_ of 0.7 to 0.8. One hour after induction, the temperature was reduced to 28°C, and expression was continued for another 4 hours. The cells were pelleted and incubated in resuspension buffer [20 mM tris-HCl, 500 mM NaCl (pH 8.0), DNase, and 1% of Triton X-100]. Cells were sonicated, and the sample was centrifuged at 30,000*g* for 30 min at 4°C. The supernatant was filtered and injected to an AKTA purifier for Ni-NTA–based purification in priming buffer [20 mM tris-HCl, 500 mM NaCl (pH 8.0), and 1% Triton X-100], and the His-tagged membrane scaffold protein (MSP) was eluted with gradient application of elution buffer [20 mM tris-HCl, 500 mM NaCl (pH 8.0), and 50 to 500 mM imidazole]. The eluted fractions were exchanged to MSP final buffer [20 mM tris-HCl and 100 mM NaCl (pH 7.4)], cleaved by TEV protease in 1:100 ratio at 4°C. The sample was then subjected to a reverse HisTrap and a final size exclusion chromatography in buffer [20 mM NaPi and 100 mM NaCl (pH 7.0)] as the final step. The protein was then aliquoted into 500 μl with a concentration of 200 μM and stored at −80°C until further use.

### BAM complex reconstitution into nanodiscs

The reconstitution of BAM into nanodiscs was done by 1,2-dimyristoyl-*sn*-glycero-3-phosphocholine (DMPC; 14:0) lipids. For MSP1D1 reconstitution, a molar ratio of BAM:MSP:DMPC:sodium cholate (1:6:312:624) was used. Samples were mixed in nanodisc buffer [25 mM NaPi and 100 mM NaCl (pH 7.0)] with Bio-Beads SM-2 adsorbent medium (Bio-Rad) for cholate and detergent (DDM) removal, mediating the nanodisc reconstitution process at room temperature on an orbital shaker overnight and forming an assembled nanodisc complex. Subsequently, the supernatant was collected and run on a size exclusion chromatography column (Superdex 200 Increase 10/300 GL).

### Surface plasmon resonance

SPR experiments were conducted on a Biacore T100 (GE Healthcare). Experiments were carried out in SPR buffer A [25 mM MES and 150 mM NaCl (pH 6.5)] at 25°C. The ligand (POTRA1, 2) (2 mg/ml) was immobilized by amine coupling to a CM5 chip surface (GE Healthcare) at a flow rate of 2 μl/min and a contact time of 1200 s. The surface of the reference channel was inactivated by amine coupling of bovine serum albumin (2 mg/ml). The interaction with SurA was measured using kinetic/affinity method from Biacore. SurA was applied to the chip at increasing concentrations (0, 0.25, 0.5, 1, 2, 4, and 10 mg/ml) at a flow of 10 μl/min and a contact time of 600 s, followed by a dissociation step of 1200 s. Chip regeneration was done with 4 M Gdm/HCl solution at a flow of 10 μl/min and a contact time of 100 s, followed by a regeneration step of 600 s with SPR buffer A. The results were analyzed using a Biacore T100 evaluation software.

Measurements of the binding affinity of SurA to the BAM complex were run at 25°C on a Biacore T200 with a STHC200M chip (XanTec) standard phosphate-buffered saline as running buffer. A Strep-tag II capture approach was used for ligand immobilization. BAM reconstituted in nanodiscs was captured at a concentration of 10 μg/ml and a flow rate of 5 μl/min until a response level of 850 resonance units (RU) was reached. For the affinity measurement, a serial dilution of SurA starting at 22 μM was prepared with 1:1 buffer:protein steps over nine points. The surface was conditioned with five buffer injections at 30 μl/min, followed by a multicycle injection of SurA with a contact time of 120 s and a dissociation time of 240 s.

Double-referenced equilibrium responses were exported with the Biacore T200 evaluation software V3.0 and analyzed with the software R 4.4.1. Simulated isotherms were calculated using the following equation

$$Y=\frac{R_{\text{max}}\times X}{K_{d}+X}$$

where *X* is the concentration, *Y* is the response, and *R*_max_ is the maximal rate constant, which was based on the immobilization estimated to be approximately 90 RU.

## References

[1] J. E. Van Wielink, J. A. Duine, How big is the periplasmic space? Trends Biochem. Sci. 15, 136–137 (1990). 2339468 10.1016/0968-0004(90)90208-s

[2] T. J. Silhavy, D. Kahne, S. Walker, The bacterial cell envelope. Cold Spring Harb. Perspect. Biol. 2, a000414 (2010). 20452953 10.1101/cshperspect.a000414 PMC2857177

[3] P. Rassam, N. A. Copeland, O. Birkholz, C. Tóth, M. Chavent, A. L. Duncan, S. J. Cross, N. G. Housden, R. Kaminska, U. Seger, D. M. Quinn, T. J. Garrod, M. S. P. Sansom, J. Piehler, C. G. Baumann, C. Kleanthous, Supramolecular assemblies underpin turnover of outer membrane proteins in bacteria. Nature 523, 333–336 (2015). 26061769 10.1038/nature14461 PMC4905513

[4] N. G. Housden, J. T. S. Hopper, N. Lukoyanova, D. Rodriguez-Larrea, J. A. Wojdyla, A. Klein, R. Kaminska, H. Bayley, H. R. Saibil, C. V. Robinson, C. Kleanthous, Intrinsically disordered protein threads through the bacterial outer-membrane porin OmpF. Science 340, 1570–1574 (2013). 23812713 10.1126/science.1237864 PMC3856478

[5] K. R. Hummels, S. P. Berry, Z. Li, A. Taguchi, J. K. Min, S. Walker, D. S. Marks, T. G. Bernhardt, Coordination of bacterial cell wall and outer membrane biosynthesis. Nature 615, 300–304 (2023). 36859542 10.1038/s41586-023-05750-0 PMC9995270

[6] H. Nikaido, Molecular basis of bacterial outer membrane permeability revisited. Microbiol. Mol. Biol. Rev. 67, 593–656 (2003). 14665678 10.1128/MMBR.67.4.593-656.2003 PMC309051

[7] N. W. Rigel, T. J. Silhavy, Making a beta-barrel: Assembly of outer membrane proteins in Gram-negative bacteria. Curr. Opin. Microbiol. 15, 189–193 (2012). 22221898 10.1016/j.mib.2011.12.007 PMC3320693

[8] J. Ude, V. Tripathi, J. M. Buyck, S. Söderholm, O. Cunrath, J. Fanous, B. Claudi, A. Egli, C. Schleberger, S. Hiller, D. Bumann, Outer membrane permeability: Antimicrobials and diverse nutrients bypass porins in Pseudomonas aeruginosa. Proc. Natl. Acad. Sci. U.S.A. 118, e2107644118 (2021). 34326266 10.1073/pnas.2107644118 PMC8346889

[9] H. Mori, K. Ito, The Sec protein-translocation pathway. Trends Microbiol. 9, 494–500 (2001). 11597451 10.1016/s0966-842x(01)02174-6

[10] G. Mas, J. Thoma, S. Hiller, The periplasmic chaperones Skp and SurA. Subcell. Biochem. 92, 169–186 (2019). 31214987 10.1007/978-3-030-18768-2_6

[11] K. Denoncin, J. Schwalm, D. Vertommen, T. J. Silhavy, J.-F. F. Collet, Dissecting the *Escherichia coli* periplasmic chaperone network using differential proteomics. Proteomics 12, 1391–1401 (2012). 22589188 10.1002/pmic.201100633 PMC3883104

[12] J. G. Sklar, T. Wu, D. Kahne, T. J. Silhavy, Defining the roles of the periplasmic chaperones SurA, Skp, and DegP in *Escherichia coli*. Genes Dev. 21, 2473–2484 (2007). 17908933 10.1101/gad.1581007 PMC1993877

[13] B. M. Burmann, C. Wang, S. Hiller, Conformation and dynamics of the periplasmic membrane-protein-chaperone complexes OmpX-Skp and tOmpA-Skp. Nat. Struct. Mol. Biol. 20, 1265–1272 (2013). 24077225 10.1038/nsmb.2677

[14] A. N. Combs, T. J. Silhavy, Periplasmic chaperones: Outer membrane biogenesis and envelope stress. Annu. Rev. Microbiol. 78, 191–211 (2024). 39008906 10.1146/annurev-micro-041522-102901 PMC12107694

[15] J. Bakelar, S. K. Buchanan, N. Noinaj, The structure of the β-barrel assembly machinery complex. Science 351, 180–186 (2016). 26744406 10.1126/science.aad3460 PMC4883095

[16] Y. Gu, H. Li, H. Dong, Y. Zeng, Z. Zhang, N. G. Paterson, P. J. Stansfeld, Z. Wang, Y. Zhang, W. Wang, C. Dong, Structural basis of outer membrane protein insertion by the BAM complex. Nature 531, 64–69 (2016). 26901871 10.1038/nature17199

[17] M. T. Doyle, H. D. Bernstein, Bacterial outer membrane proteins assemble via asymmetric interactions with the BamA β-barrel. Nat. Commun. 10, 3358 (2019). 31350400 10.1038/s41467-019-11230-9 PMC6659671

[18] M. G. Iadanza, A. J. Higgins, B. Schiffrin, A. N. Calabrese, D. J. Brockwell, A. E. Ashcroft, S. E. Radford, N. A. Ranson, Lateral opening in the intact β-barrel assembly machinery captured by cryo-EM. Nat. Commun. 7, 12865 (2016). 27686148 10.1038/ncomms12865 PMC5056442

[19] A. Konovalova, D. E. Kahne, T. J. Silhavy, Outer membrane biogenesis. Annu. Rev. Microbiol. 71, 539–556 (2017). 28886680 10.1146/annurev-micro-090816-093754 PMC5778897

[20] J. Lee, D. Tomasek, T. M. Santos, M. D. May, I. Meuskens, D. Kahne, Formation of a β-barrel membrane protein is catalyzed by the interior surface of the assembly machine protein BamA. eLife 8, e49787 (2019). 31724945 10.7554/eLife.49787 PMC6887485

[21] D. Tomasek, S. Rawson, J. Lee, J. S. Wzorek, S. C. Harrison, Z. Li, D. Kahne, Structure of a nascent membrane protein as it folds on the BAM complex. Nature 583, 473–478 (2020). 32528179 10.1038/s41586-020-2370-1 PMC7367713

[22] M. T. Doyle, J. R. Jimah, T. Dowdy, S. I. Ohlemacher, M. Larion, J. E. Hinshaw, H. D. Bernstein, Cryo-EM structures reveal multiple stages of bacterial outer membrane protein folding. Cell 185, 1143–1156.e13 (2022). 35294859 10.1016/j.cell.2022.02.016 PMC8985213

[23] R. Wu, J. W. Bakelar, K. Lundquist, Z. Zhang, K. M. Kuo, D. Ryoo, Y. T. Pang, C. Sun, T. White, T. Klose, W. Jiang, J. C. Gumbart, N. Noinaj, Plasticity within the barrel domain of BamA mediates a hybrid-barrel mechanism by BAM. Nat. Commun. 12, 7131 (2021). 34880256 10.1038/s41467-021-27449-4 PMC8655018

[24] C. Shen, S. Chang, Q. Luo, K. C. Chan, Z. Zhang, B. Luo, T. Xie, G. Lu, X. Zhu, X. Wei, C. Dong, R. Zhou, X. Zhang, X. Tang, H. Dong, Structural basis of BAM-mediated outer membrane β-barrel protein assembly. Nature 617, 185–193 (2023). 37100902 10.1038/s41586-023-05988-8

[25] H. Kaur, J. B. Hartmann, R. P. Jakob, M. Zahn, I. Zimmermann, T. Maier, M. A. Seeger, S. Hiller, Identification of conformation-selective nanobodies against the membrane protein insertase BamA by an integrated structural biology approach. J. Biomol. NMR 73, 375–384 (2019). 31073665 10.1007/s10858-019-00250-8

[26] L. Xiao, L. Han, B. Li, M. Zhang, H. Zhou, Q. Luo, X. Zhang, Y. Huang, Structures of the β-barrel assembly machine recognizing outer membrane protein substrates. FASEB J. 35, e21207 (2021). 33368572 10.1096/fj.202001443RR

[27] Y. Imai, K. J. Meyer, A. Iinishi, Q. Favre-Godal, R. Green, S. Manuse, M. Caboni, M. Mori, S. Niles, M. Ghiglieri, C. Honrao, X. Ma, J. J. Guo, A. Makriyannis, L. Linares-Otoya, N. Böhringer, Z. G. Wuisan, H. Kaur, R. Wu, A. Mateus, A. Typas, M. M. Savitski, J. L. Espinoza, A. O’Rourke, K. E. Nelson, S. Hiller, N. Noinaj, T. F. Schäberle, A. D’Onofrio, K. Lewis, A new antibiotic selectively kills Gram-negative pathogens. Nature 576, 459–464 (2019). 31747680 10.1038/s41586-019-1791-1 PMC7188312

[28] H. Kaur, R. P. Jakob, J. K. Marzinek, R. Green, Y. Imai, J. R. Bolla, E. Agustoni, C. V. Robinson, P. J. Bond, K. Lewis, T. Maier, S. Hiller, The antibiotic darobactin mimics a β-strand to inhibit outer membrane insertase. Nature 593, 125–129 (2021). 33854236 10.1038/s41586-021-03455-w

[29] R. D. Miller, A. Iinishi, S. M. Modaresi, B.-K. Yoo, T. D. Curtis, P. J. Lariviere, L. Liang, S. Son, S. Nicolau, R. Bargabos, M. Morrissette, M. F. Gates, N. Pitt, R. P. Jakob, P. Rath, T. Maier, A. G. Malyutin, J. T. Kaiser, S. Niles, B. Karavas, M. Ghiglieri, S. E. J. Bowman, D. C. Rees, S. Hiller, K. Lewis, Computational identification of a systemic antibiotic for Gram-negative bacteria. Nat. Microbiol. 7, 1661–1672 (2022). 36163500 10.1038/s41564-022-01227-4 PMC10155127

[30] F. Gruss, F. Zähringer, R. P. Jakob, B. M. Burmann, S. Hiller, T. Maier, The structural basis of autotransporter translocation by TamA. Nat. Struct. Mol. Biol. 20, 1318–1320 (2013). 24056943 10.1038/nsmb.2689

[31] S. W. Lazar, R. Kolter, SurA assists the folding of Escherichia coli outer membrane proteins. J. Bacteriol. 178, 1770–1773 (1996). 8626309 10.1128/jb.178.6.1770-1773.1996 PMC177866

[32] E. Bitto, D. B. McKay, Crystallographic structure of SurA, a molecular chaperone that facilitates folding of outer membrane porins. Structure 10, 1489–1498 (2002). 12429090 10.1016/s0969-2126(02)00877-8

[33] G. R. Soltes, J. Schwalm, D. P. Ricci, T. J. Silhavy, The activity of *Escherichia coli* chaperone SurA is regulated by conformational changes involving a parvulin domain. J. Bacteriol. 198, 921–929 (2016). 26728192 10.1128/JB.00889-15 PMC4772605

[34] B. Schiffrin, J. M. Machin, T. K. Karamanos, A. Zhuravleva, D. J. Brockwell, S. E. Radford, A. N. Calabrese, Dynamic interplay between the periplasmic chaperone SurA and the BAM complex in outer membrane protein folding. Commun. Biol. 5, 560 (2022). 35676411 10.1038/s42003-022-03502-w PMC9177699

[35] K. L. Fenn, J. E. Horne, J. A. Crossley, N. Böhringer, R. J. Horne, T. F. Schäberle, A. N. Calabrese, S. E. Radford, N. A. Ranson, Outer membrane protein assembly mediated by BAM-SurA complexes. Nat. Commun. 15, 7612 (2024). 39218969 10.1038/s41467-024-51358-x PMC11366764

[36] M. Gao, D. Nakajima An, J. Skolnick, Deep learning-driven insights into super protein complexes for outer membrane protein biogenesis in bacteria. eLife 11, e82885 (2022). 36576775 10.7554/eLife.82885 PMC9797188

[37] D. Bennion, E. S. Charlson, E. Coon, R. Misra, Dissection of β-barrel outer membrane protein assembly pathways through characterizing BamA POTRA 1 mutants of *Escherichia coli*. Mol. Microbiol. 77, 1153–1171 (2010). 20598079 10.1111/j.1365-2958.2010.07280.x PMC2975826

[38] X. Xu, S. Wang, Y.-X. X. Hu, D. B. McKay, The periplasmic bacterial molecular chaperone SurA adapts its structure to bind peptides in different conformations to assert a sequence preference for aromatic residues. J. Mol. Biol. 373, 367–381 (2007). 17825319 10.1016/j.jmb.2007.07.069 PMC2040117

[39] A. Schmidt, K. Kochanowski, S. Vedelaar, E. Ahrné, B. Volkmer, L. Callipo, K. Knoops, M. Bauer, R. Aebersold, M. Heinemann, The quantitative and condition-dependent *Escherichia coli* proteome. Nat. Biotechnol. 34, 104–110 (2016). 26641532 10.1038/nbt.3418 PMC4888949

[40] G. Mas, B. M. Burmann, T. Sharpe, B. Claudi, D. Bumann, S. Hiller, Regulation of chaperone function by coupled folding and oligomerization. Sci. Adv. 6, eabc5822 (2020). 33087350 10.1126/sciadv.abc5822 PMC7577714

[41] I. P. Korndörfer, M. K. Dommel, A. Skerra, Structure of the periplasmic chaperone Skp suggests functional similarity with cytosolic chaperones despite differing architecture. Nat. Struct. Mol. Biol. 11, 1015–1020 (2004). 15361861 10.1038/nsmb828

[42] B. Schiffrin, A. N. Calabrese, A. J. Higgins, J. R. Humes, A. E. Ashcroft, A. C. Kalli, D. J. Brockwell, S. E. Radford, Effects of periplasmic chaperones and membrane thickness on BamA-catalyzed outer-membrane protein folding. J. Mol. Biol. 429, 3776–3792 (2017). 28919234 10.1016/j.jmb.2017.09.008 PMC5692476

[43] A. Punjani, J. L. Rubinstein, D. J. Fleet, M. A. Brubaker, cryoSPARC: Algorithms for rapid unsupervised cryo-EM structure determination. Nat. Methods 14, 290–296 (2017). 28165473 10.1038/nmeth.4169

[44] R. Sanchez-Garcia, J. Gomez-Blanco, A. Cuervo, J. M. Carazo, C. O. S. Sorzano, J. Vargas, DeepEMhancer: A deep learning solution for cryo-EM volume post-processing. Commun. Biol. 4, 874 (2021). 34267316 10.1038/s42003-021-02399-1 PMC8282847

[45] E. C. Meng, T. D. Goddard, E. F. Pettersen, G. S. Couch, Z. J. Pearson, J. H. Morris, T. E. Ferrin, UCSF ChimeraX: Tools for structure building and analysis. Protein Sci. 32, e4792 (2023). 37774136 10.1002/pro.4792 PMC10588335

[46] M. Varadi, D. Bertoni, P. Magana, U. Paramval, I. Pidruchna, M. Radhakrishnan, M. Tsenkov, S. Nair, M. Mirdita, J. Yeo, O. Kovalevskiy, K. Tunyasuvunakool, A. Laydon, A. Žídek, H. Tomlinson, D. Hariharan, J. Abrahamson, T. Green, J. Jumper, E. Birney, M. Steinegger, D. Hassabis, S. Velankar, AlphaFold Protein Structure Database in 2024: Providing structure coverage for over 214 million protein sequences. Nucleic Acids Res. 52, D368–D375 (2024). 37933859 10.1093/nar/gkad1011 PMC10767828

[47] M. Varadi, S. Anyango, M. Deshpande, S. Nair, C. Natassia, G. Yordanova, D. Yuan, O. Stroe, G. Wood, A. Laydon, A. Žídek, T. Green, K. Tunyasuvunakool, S. Petersen, J. Jumper, E. Clancy, R. Green, A. Vora, M. Lutfi, M. Figurnov, A. Cowie, N. Hobbs, P. Kohli, G. Kleywegt, E. Birney, D. Hassabis, S. Velankar, AlphaFold Protein Structure Database: Massively expanding the structural coverage of protein-sequence space with high-accuracy models. Nucleic Acids Res. 50, D439–D444 (2022). 34791371 10.1093/nar/gkab1061 PMC8728224

[48] T. I. Croll, ISOLDE: A physically realistic environment for model building into low-resolution electron-density maps. Acta Crystallogr. D Struct. Biol. 74, 519–530 (2018). 29872003 10.1107/S2059798318002425 PMC6096486

[49] P. Emsley, K. Cowtan, Coot: Model-building tools for molecular graphics. Acta Crystallogr. D Biol. Crystallogr. 60, 2126–2132 (2004). 15572765 10.1107/S0907444904019158

[50] P. D. Adams, R. W. Grosse-Kunstleve, L. W. Hung, T. R. Ioerger, A. J. McCoy, N. W. Moriarty, R. J. Read, J. C. Sacchettini, N. K. Sauter, T. C. Terwilliger, PHENIX: Building new software for automated crystallographic structure determination. Acta Crystallogr. D Biol. Crystallogr. 58, 1948–1954 (2002). 12393927 10.1107/s0907444902016657

[51] P. V. Afonine, B. K. Poon, R. J. Read, O. V. Sobolev, T. C. Terwilliger, A. Urzhumtsev, P. D. Adams, Real-space refinement in PHENIX for cryo-EM and crystallography. Acta Crystallogr. D Struct. Biol. 74, 531–544 (2018). 29872004 10.1107/S2059798318006551 PMC6096492

[52] V. B. Chen, W. B. Arendall, J. J. Headd, D. A. Keedy, R. M. Immormino, G. J. Kapral, L. W. Murray, J. S. Richardson, D. C. Richardson, MolProbity: All-atom structure validation for macromolecular crystallography. Acta Crystallogr. D Biol. Crystallogr. 66, 12–21 (2010). 20057044 10.1107/S0907444909042073 PMC2803126

[53] P. V. Afonine, B. P. Klaholz, N. W. Moriarty, B. K. Poon, O. V. Sobolev, T. C. Terwilliger, P. D. Adams, A. Urzhumtsev, New tools for the analysis and validation of cryo-EM maps and atomic models. Acta Crystallogr. D Struct. Biol. 74, 814–840 (2018). 30198894 10.1107/S2059798318009324 PMC6130467

[54] C. J. Williams, J. J. Headd, N. W. Moriarty, M. G. Prisant, L. L. Videau, L. N. Deis, V. Verma, D. A. Keedy, B. J. Hintze, V. B. Chen, S. Jain, S. M. Lewis, W. B. Arendall, J. Snoeyink, P. D. Adams, S. C. Lovell, J. S. Richardson, D. C. Richardson, MolProbity: More and better reference data for improved all-atom structure validation. Protein Sci. 27, 293–315 (2018). 29067766 10.1002/pro.3330 PMC5734394

