# Supplementary material for: Architecture and conformational dynamics of the BAM-SurA holo insertase complex
Source: Sci Adv. 2025 Apr 4;11(14):eads6094. doi: 10.1126/sciadv.ads6094 (PMC11970506; doi:10.1126/sciadv.ads6094)
Supplement: Supplementary file 1 — Supplementary Text Figs. S1 to S10 Table S1 Legend for movie S1 [file sciadv.ads6094_sm.pdf]

Science Advances
AAAS

Supplementary Materials for

Architecture and conformational dynamics of the BAM-SurA holo insertase complex

Philippe A. Lehner *et al.*

Corresponding author: Sebastian Hiller, sebastian.hiller@unibas.ch

Sci. Adv. **11**, eads6094 (2025)
DOI: 10.1126/sciadv.ads6094

The PDF file includes:

Supplementary Text
Figs. S1 to S10
Table S1
Legend for movie S1

Other Supplementary Material for this manuscript includes the following:

Movie S1

Supplementary Text

Cryo-EM workflow (Figure S3)

A total of 68,464 movies for BAM–SurA were recorded, as two separate datasets. Below, we will discuss the steps of data processing on the example of the larger dataset with 58,464 movies. Out of these, 3,233 movies were excluded due to poor local sample quality, leaving 55,231 movies for particle picking. Particle picking was conducted with the blob-picker tool, followed by template picking, yielding around 500 picks per micrograph (**Fig. S3A**). The picks were combined, and duplicates were removed, resulting in a total of 11,181,244 initial particles. 2D classification was applied to these initial particles (**Fig. S3B**), during which the particles were still binned four times. Additionally, heterogeneous refinement was applied, using good classes and artifact classes. This step thus removed low-resolution particles, carbon edges and impurities from the dataset, resulting in a dataset of 3,191,037 high-quality particles.

Next, the particles were re-extracted without binning, and 3D classifications were applied to separate different conformational states within the dataset (**Fig. S3C**). At this stage, approximately one-fourth (750,151 particles) of all particles were excluded because they either lacked visible density for SurA or showed low-resolution density in the region of interest. The remaining particles were classified based on swing-in and swing-out conformations, with and without BamC present. To achieve high-resolution structures, in a final step only high-resolution particles were selected, resulting in an overall final dataset of 1,673,309 particles.

In summary, it can be concluded that, during the iterative process of particle curation from cryo-EM data, only i) artifacts from data recording, ii) particles in grid areas with low local resolution, or iii) particles without bound SurA were excluded from the workflow. The data set thus retains a statistical representation of the conformational ensemble of the BAM–SurA holo insertase complex. The final particle specifically resolved the swing-in and swing-out conformations, which represent the endpoints of a continuous swing motion, as demonstrated by 3D classification and 3D variability analysis.

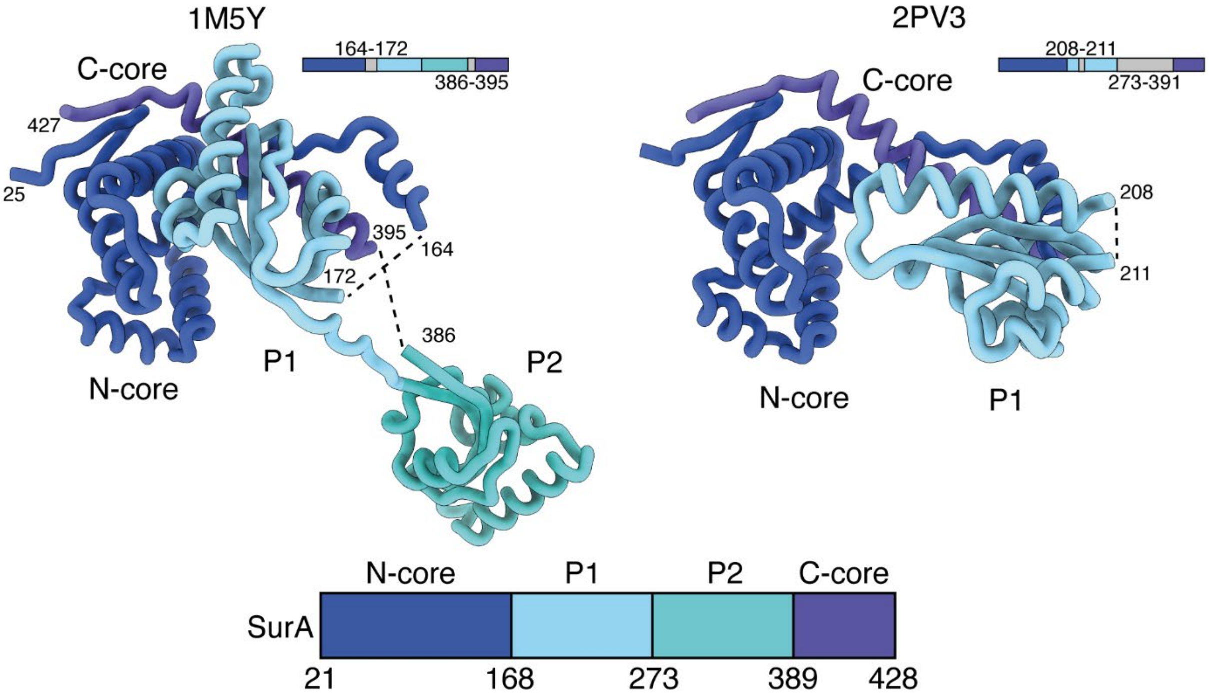

Fig. S1. Structures of SurA. Previously published crystal structures of SurA with different subdomains colored in shades of blue. Left: PDB: 1M5Y (*32*); right: PDB: 2PV3 (*37*). Below the structures, the domain architecture of SurA is shown. For each of the structures, the experimentally unresolved parts are indicated by dashed lines in the structure and by grey boxes on the sequence.

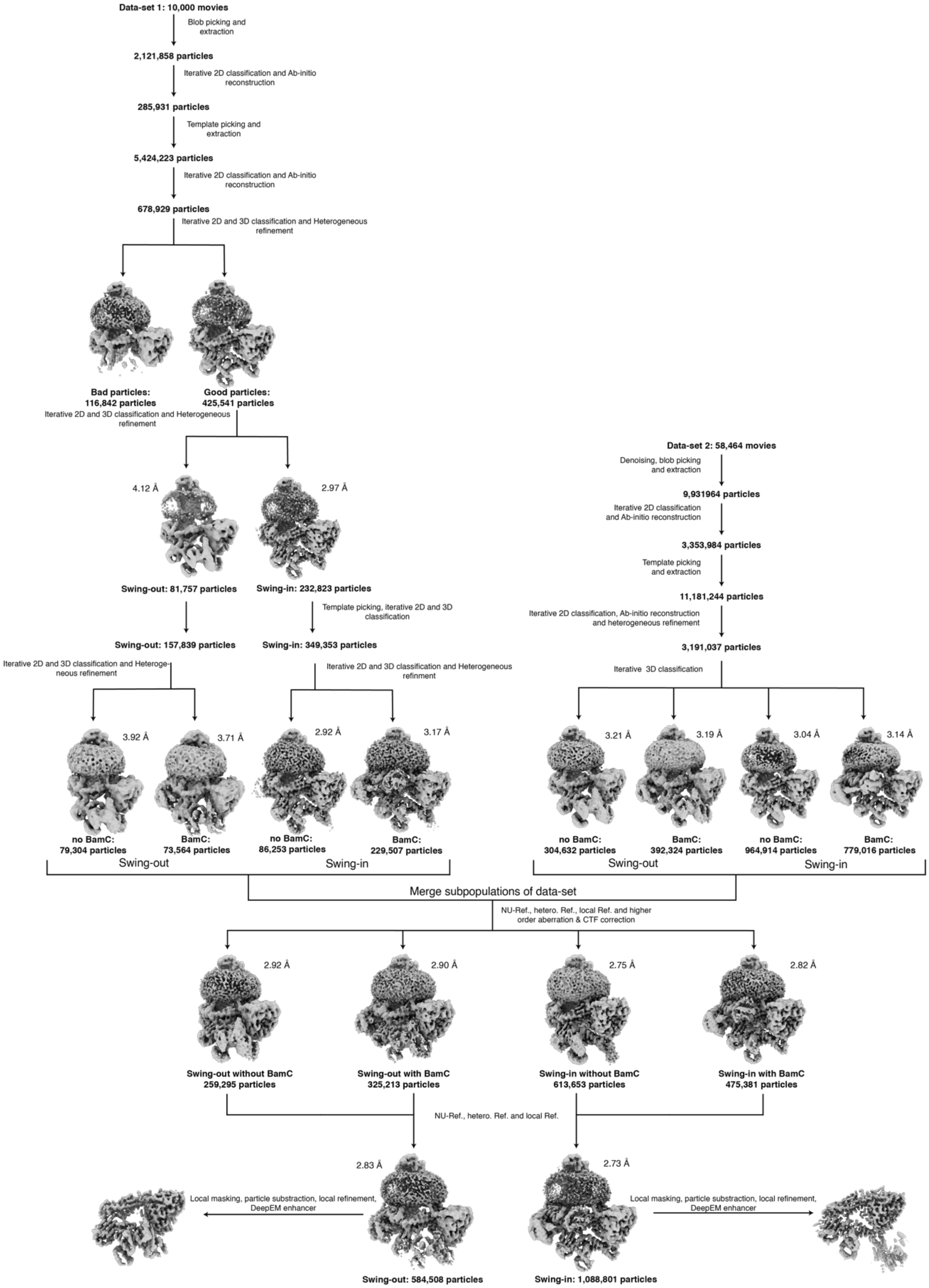

Fig. S2. Cryo-EM workflow for the structure determination of BAM–SurA. Schematic overview of the processing workflow. See Materials and Methods for details.

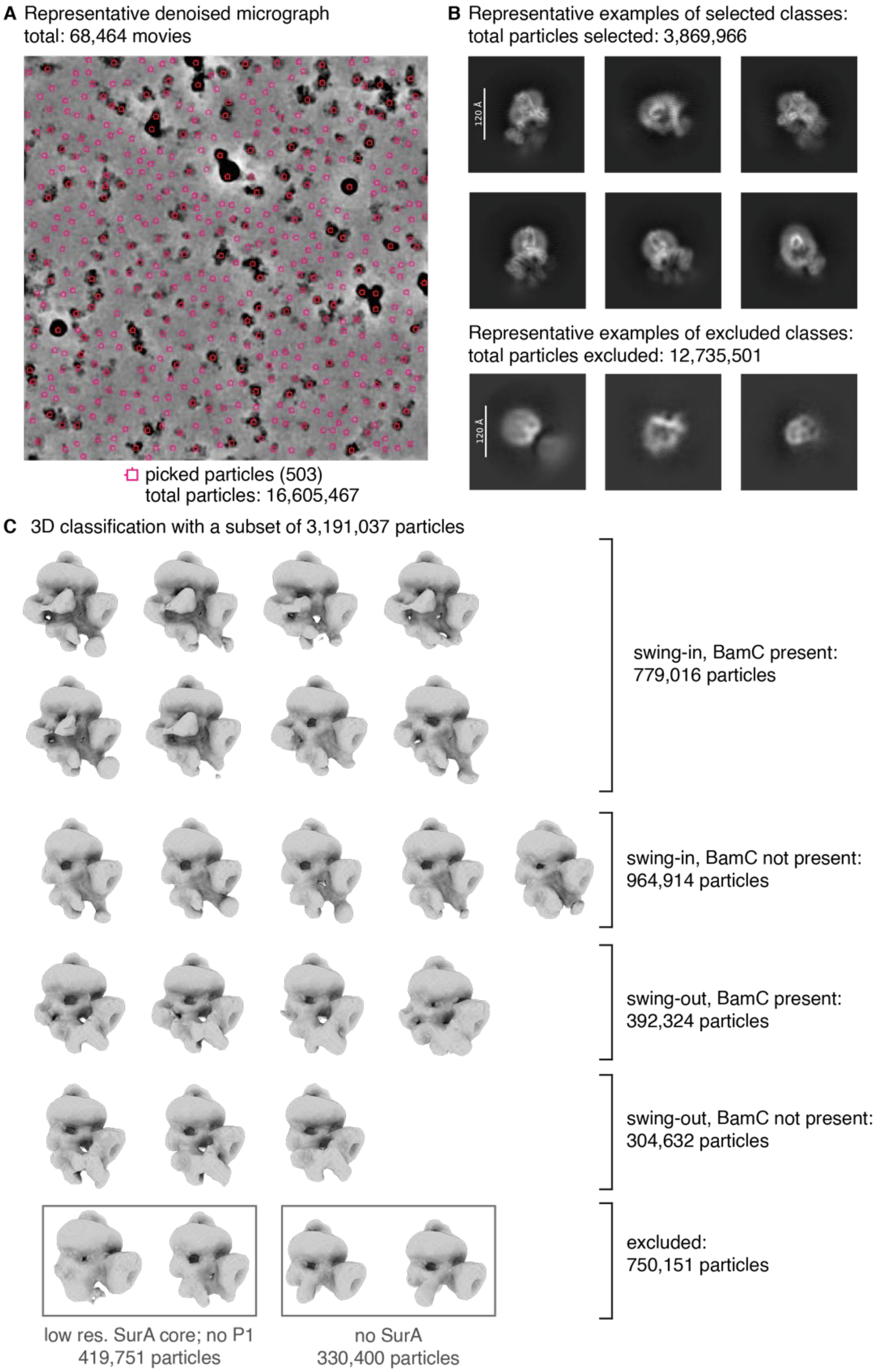

Fig. S3. Cryo-EM particle selection and exclusion. A Representative example of a denoised micrograph showing particle picks (pink square) using the template-picker. **B** Representative example classes which were selected or excluded during iterative 2D classification. **C** Cryo-EM maps of 3D classified classes sorted according to the different states observed using a subset of 3,191,037.

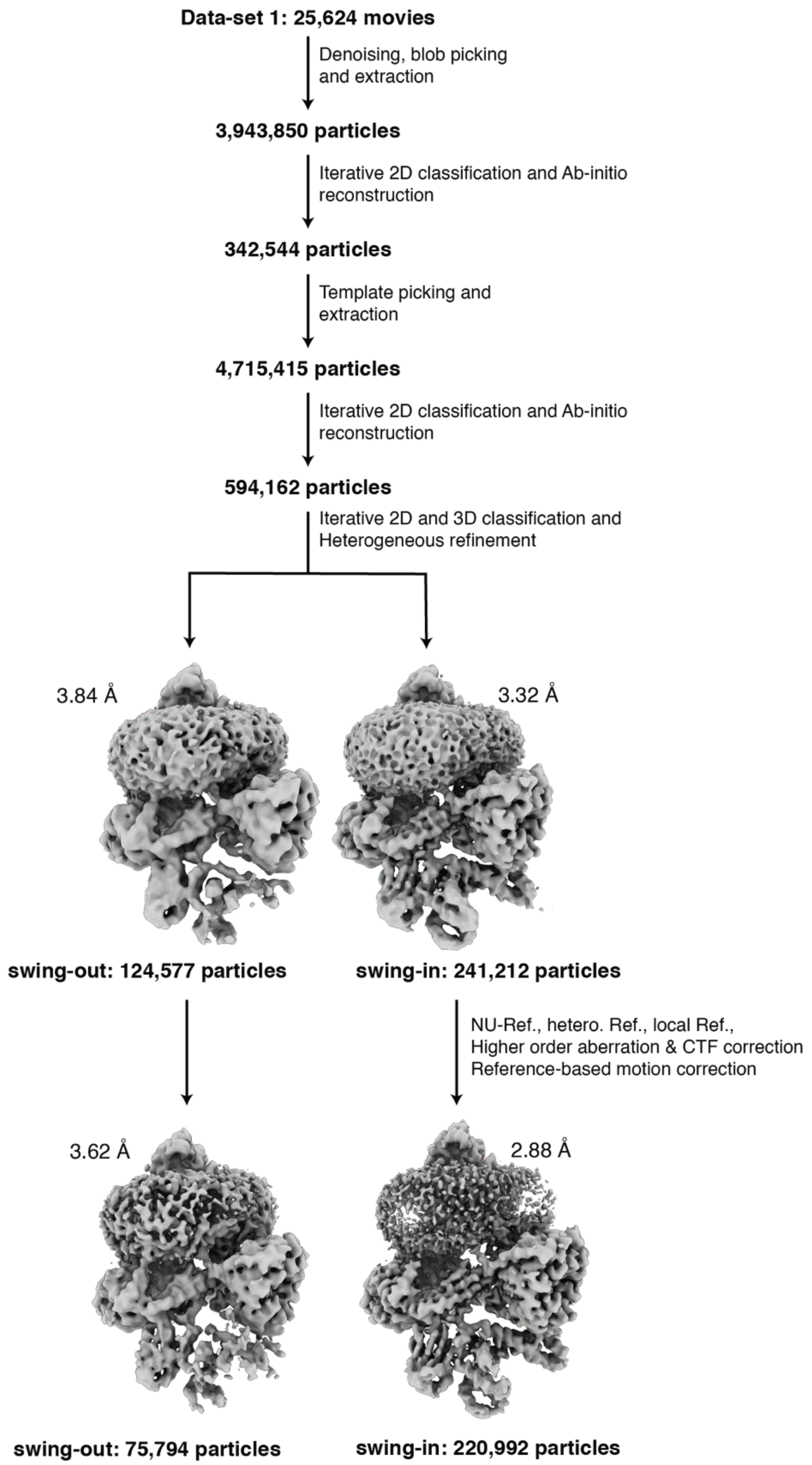

Fig. S4. Cryo-EM workflow for the structure determination of BAM–SurA in presence of darobactin A. Schematic overview of the processing workflow. See Materials and Methods for details.

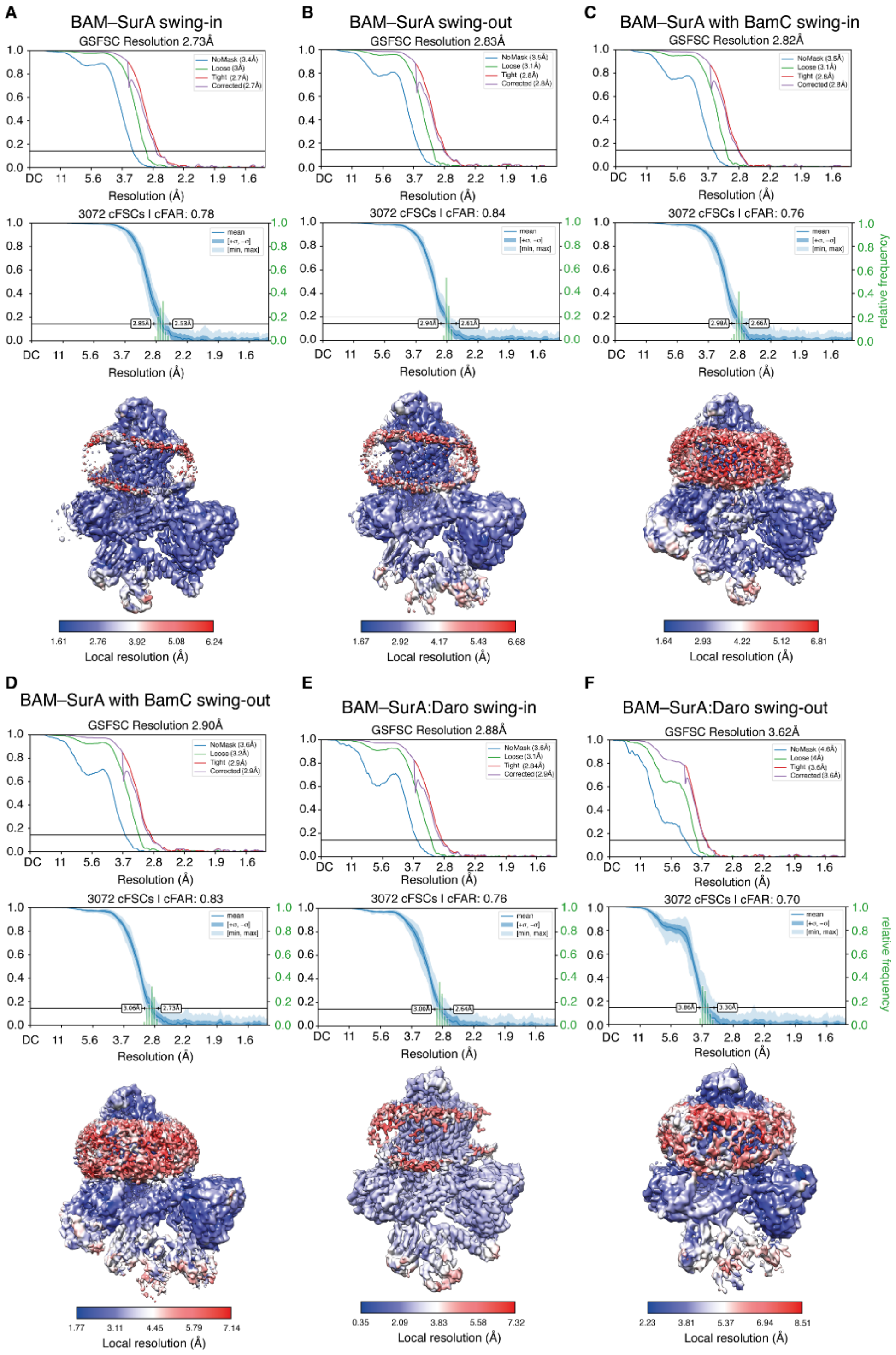

Fig. S5. Cryo-EM global and directional FSC resolution of all resolved BAM–SurA states. Gold standard Fourier shell correlation (GSFSC) curves and conical Fourier shell correlation (cFSC) | conical FSC area ratio (cFAR) plots along with local resolution maps (blue: low, red: high) are shown for panels **A**–**F**. **A** BAM–SurA swing-in state. **B** BAM–SurA swing-out state. **C** BAM–SurA swing-in state with resolved full-length BamC. **D** BAM–SurA swing-out state with partially resolved full-length BamC. **E** BAM–SurA–darobactin swing-in state. **F** BAM–SurA–darobactin swing-out state.

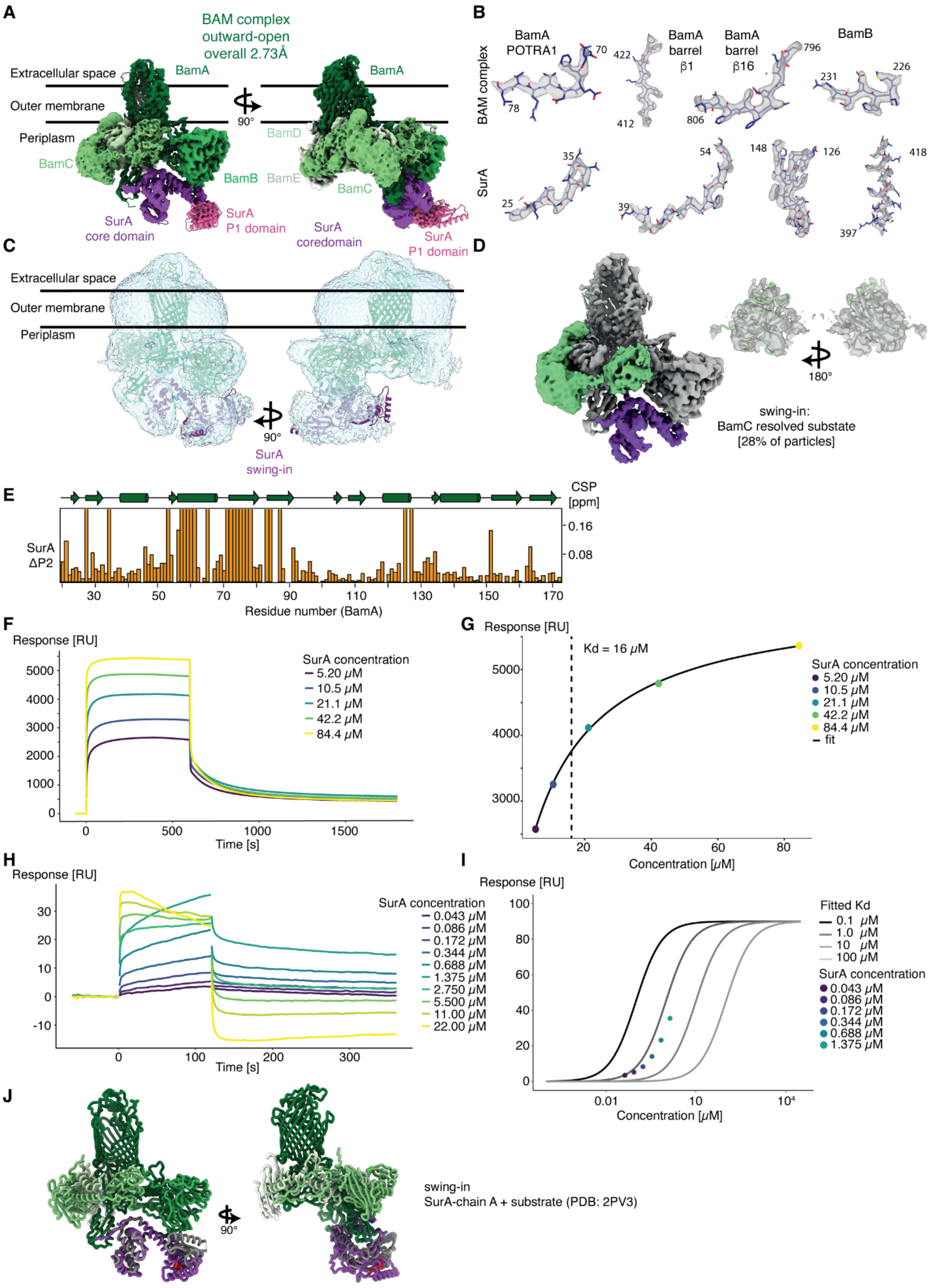

A
BAM complex
outward-open
overall 2.73Å
Extracellular space
Outer membrane
Periplasm
BamA
BamD
BamC
BamB
BamE
SurA
core domain
SurA
P1 domain
SurA
coredomain
90°
B
BAM complex
SurA
BamA
POTRA1
BamA
barrel
β1
BamA
barrel
β16
BamB
C
Extracellular space
Outer membrane
Periplasm
90°
SurA
swing-in
D
180°
swing-in:
BamC resolved substate
[28% of particles]
E
SurA
ΔP2
CSP
[ppm]
Residue number (BamA)
F
Response [RU]
Time [s]
SurA concentration
5.20 μM
10.5 μM
21.1 μM
42.2 μM
84.4 μM
G
Response [RU]
Kd = 16 μM
Concentration [μM]
SurA concentration
5.20 μM
10.5 μM
21.1 μM
42.2 μM
84.4 μM
fit
H
Response [RU]
Time [s]
SurA concentration
0.043 μM
0.086 μM
0.172 μM
0.344 μM
0.688 μM
1.375 μM
2.750 μM
5.500 μM
11.00 μM
22.00 μM
I
Response [RU]
Concentration [μM]
Fitted Kd
0.1 μM
1.0 μM
10 μM
100 μM
SurA concentration
0.043 μM
0.086 μM
0.172 μM
0.344 μM
0.688 μM
1.375 μM
J
90°
swing-in
SurA-chain A + substrate (PDB: 2PV3)

Fig. S6. Structural and biophysical analysis of the BAM–SurA *holo* insertase complex. **A** Composite map at accordingly adjusted contour level of the swing-in conformations with the fitted structure model. The map and model are shown in a gradient of greens for the BAM components. SurA is shown in purple. **B** Examples of modeled chains from different regions of the structure, with the first and last amino acid number labeled, shown individually within the experimentally determined cryo-EM density (grey, transparent surface). **C** High contour level map for the swing-in state with the corresponding model, showing the presence of the flexible SurA-P1 and P2 domains. **D** Cryo-EM density with individual adjusted contour levels of each domain showing the presence of the full-length BamC domain in the swing-in state in 28% of all particles. BamC is highlighted in green. The density corresponding to SurA is depicted in purple. A close-up of the density for BamC C-terminal helix grip-domain with the corresponding model is shown. **E** Chemical shift perturbations (CSPs, in ppm) of POTRA1, 2 residues in the presence of SurAΔP2 plotted against the residue number with secondary structure of POTRA1, 2 indicated above in green. **F** SPR sensorgrams for POTRA1, 2 with increasing concentrations of the analyte SurA (5.20 – 84.4 μM). **G** SPR binding isotherm based on steady state signal of **F**, with a determined Kd of 16 μM. **H** SPR sensorgrams showing a multi cycle run over time for BAM with increasing concentrations of SurA (0.043 – 22 μM). **I** SPR binding isotherm based on steady state signal of concentration 0.043 – 1.375 μM. Cycles with higher SurA concentration were excluded due to unspecific binding to the reference channel. Binding curves are calculated for a Kd of 0.1, 1, 10 and 100 μM with an estimated R_{max} of 90 RU. The analysis suggests a binding affinity in the low micromolar range. **J** BAM–SurA structure in the swing-in conformation overlayed with the monomer from a previously determined peptide substrate-bound crystal structure of SurA (PDB: 2PV3, grey).

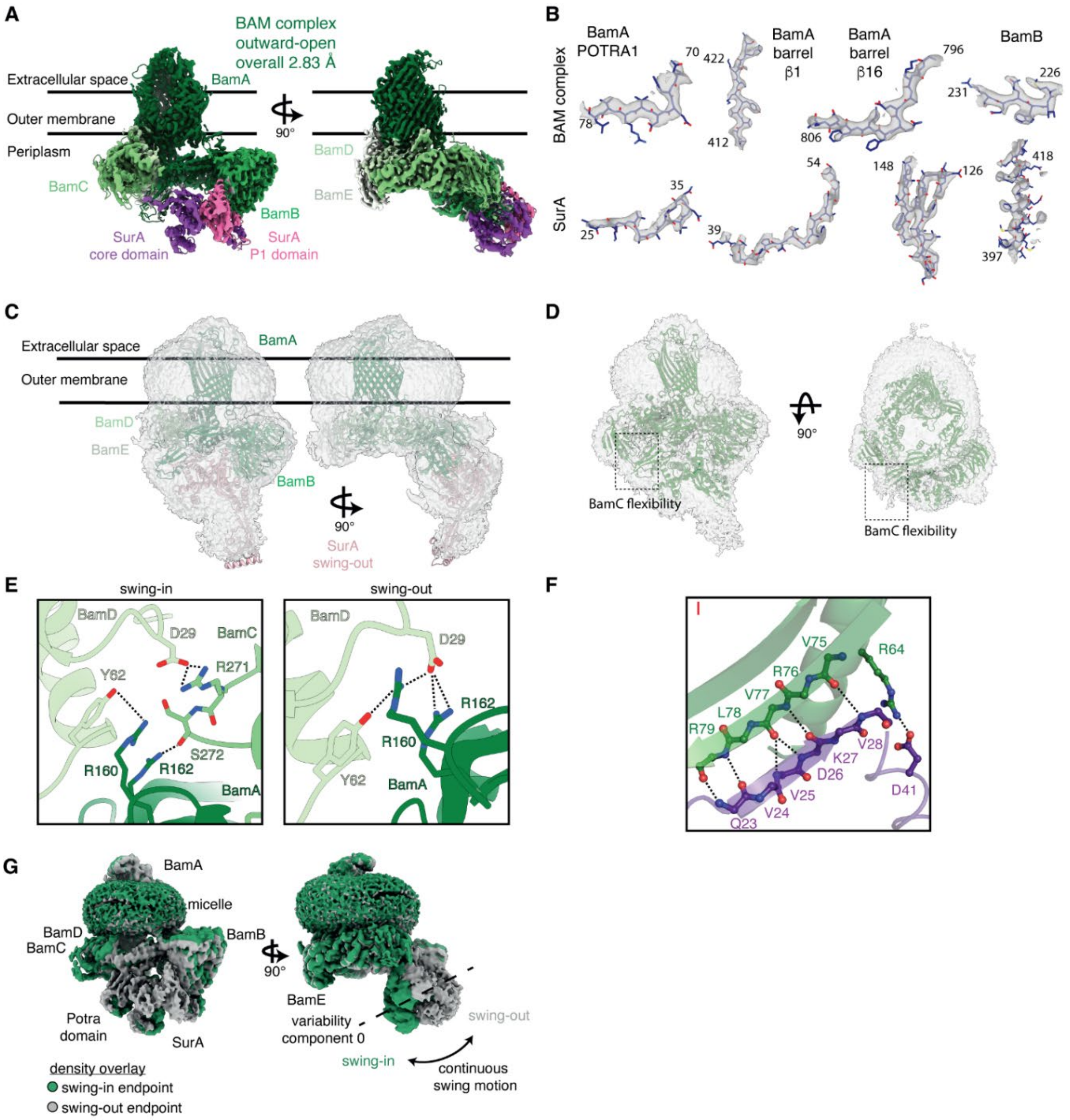

Fig. S7. Structural analysis of BAM–SurA swing-out. A Composite map at accordingly adjusted contour level of the swing-out conformations with the fitted structure model. The map and model are shown in a gradient of greens for the BAM components. SurA is shown in purple. **B** Examples of modeled chains from different regions of the structure, with the first and last amino acid number labeled, shown individually within the experimentally determined cryo-EM density (grey, transparent surface). **C** High contour level map for the swing-out state with the corresponding model, showing the presence of the flexible SurA-P2 domain. **D** High contour-level map with the model of BAM–SurA swing-out in green, showing the partial presence of BamC. **E** Close-up of the key interactions of the interface between BamD (light green), BamA (dark green) and BamC (green). Left: swing-in state; Right: swing-out state. **F** Close-up of the interface I of SurA (purple) with BamA POTRA1 (green). **G** Overlay of the density of the swing-in (green) and swing-out (grey) state, highlighting the swing motion of SurA together with POTRA1.

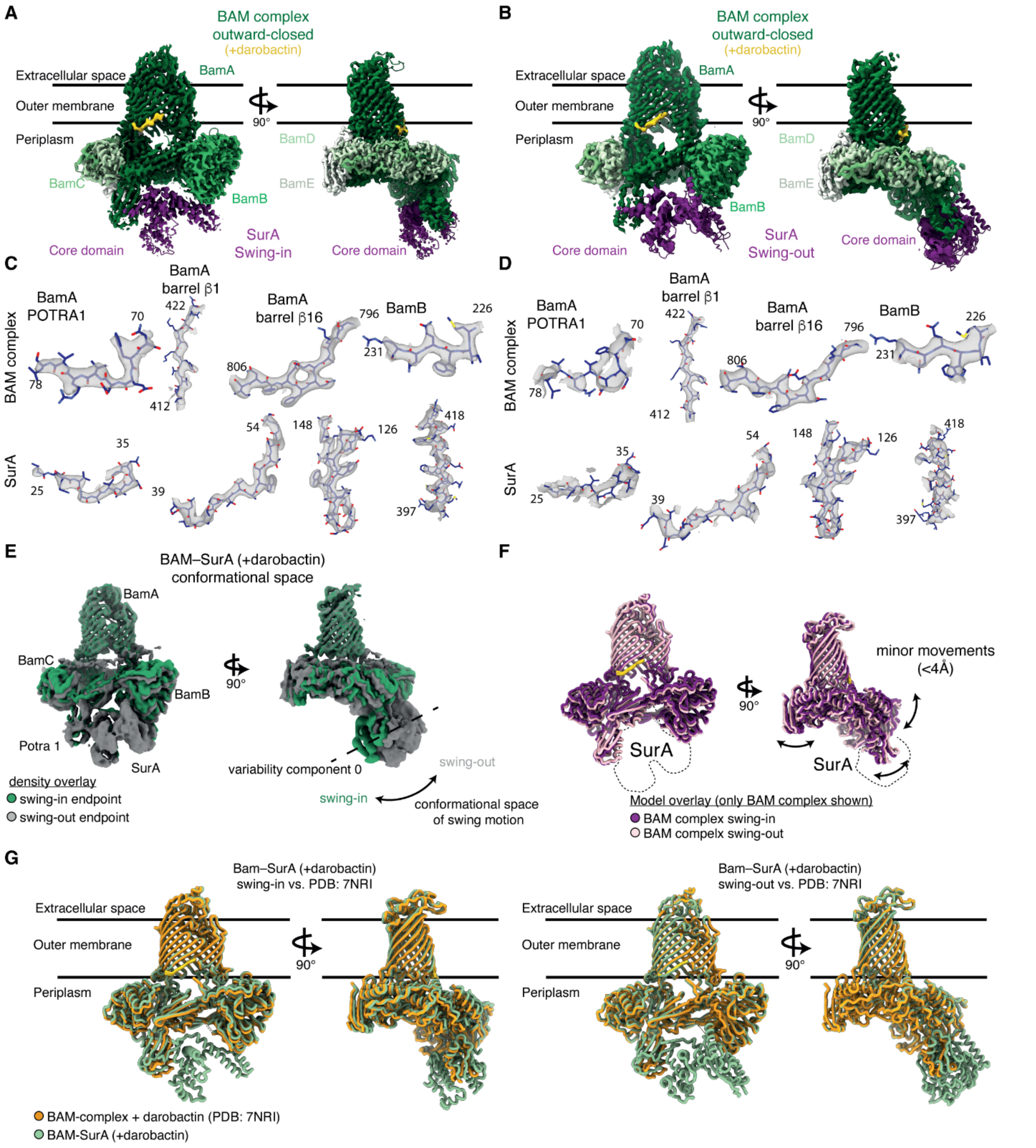

Fig. S8. Structure determination and dynamics comparison of BAM–SurA in presence of darobactin. A Composite map at accordingly adjusted contour level of the swing-in conformations with the fitted structure model. The map and model are shown in a gradient of greens for the BAM components. SurA is shown in purple and darobactin in yellow. **B** Composite map at accordingly adjusted contour level of the swing-out conformations with the fitted structural model. The map and model are shown in a gradient of greens for the BAM components. SurA is shown in purple and darobactin A in yellow. **C–D** Examples of modeled chains from different regions of the structures, with the first and last amino acid number labeled, shown individually

within the experimentally determined cryo-EM map (grey, transparent surface). **E** Overlay of the density of the swing-in (green) and swing-out (grey) state, highlighting the swing-motion of surA together with POTRA1. **F** Overlay of the structures of the swing-in (purple) and swing-out (pink) state, highlighting the alignment of the BAM components **G** Alignment of the structure of BAM–SurA–darobactin (green) with the previously determined structure of BAM–darobactin (PDB: 7NRI, orange) on the left side for the swing-in state – on the right side for the swing-out state.

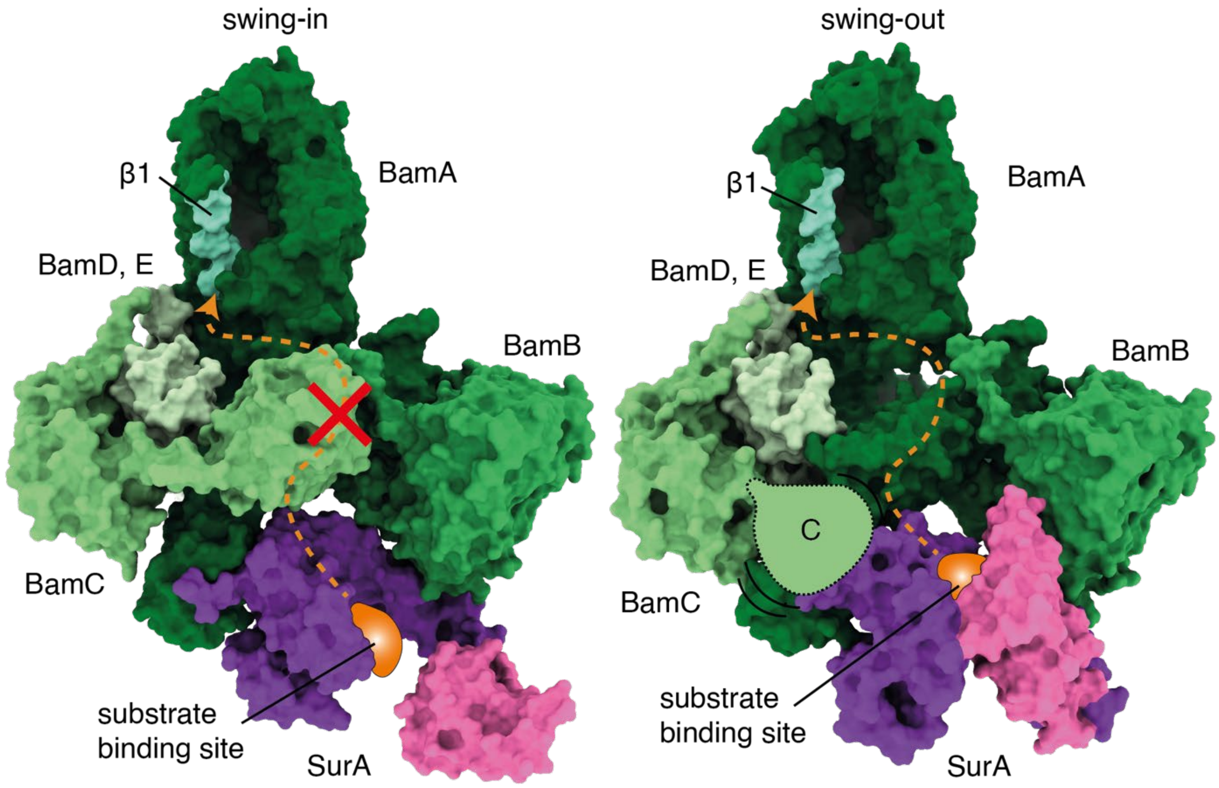

Fig. S9. Structural analysis of a potential substrate pathway from the SurA binding site to the lateral gate. BAM is shown in shades of green, SurA core in purple and its flexible P1 domain in pink. β-strand 1 of BamA forming one side of the lateral gate is highlighted in blue. The approximate substrate binding side of an unfolded substrate is highlighted in orange. The binding side is adapted from 8QPW.pdb. A potential path for the substrate from the binding side to the lateral gate is highlighted by an orange arrow. Left: swing-in state; Right: swing-out state. BamC is only fully resolved in the swing-in state. In the swing-out state the C-terminal helix grip domain of BamC is shown as a green sphere, to show its flexibility and detaching from POTRA2 and BamD, leading to an opening of a potential pathway for incoming substrate.

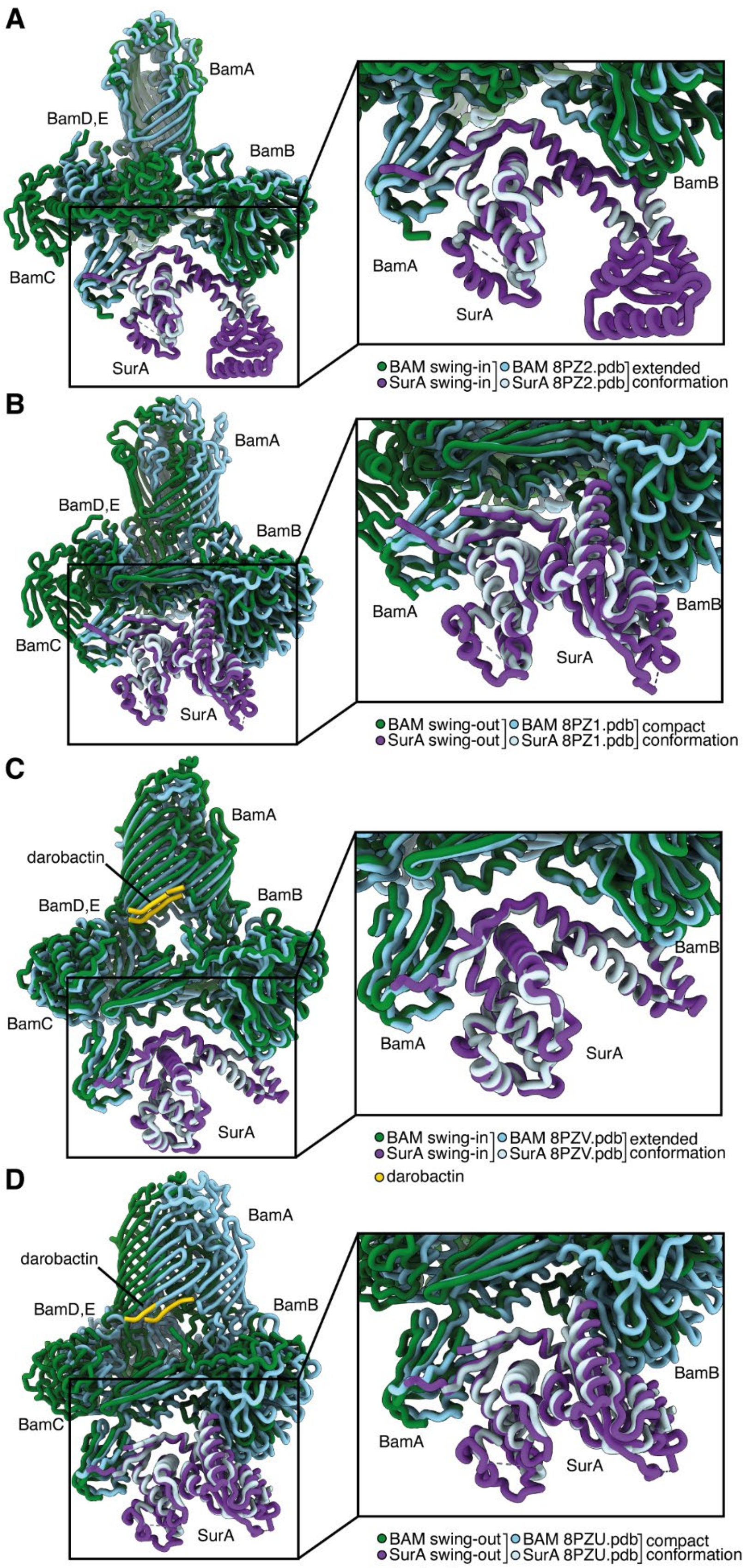
A
BamA
BamD,E
BamB
BamC
SurA
BamB
BamA
SurA
BAM swing-in
SurA swing-in
BAM 8PZ2.pdb
SurA 8PZ2.pdb
extended conformation
B
BamA
BamD,E
BamB
BamC
SurA
BamA
BamB
SurA
BAM swing-out
SurA swing-out
BAM 8PZ1.pdb
SurA 8PZ1.pdb
compact conformation
C
BamA
darobactin
BamD,E
BamB
BamC
SurA
BamB
BamA
SurA
BAM swing-in
SurA swing-in
BAM 8PZV.pdb
SurA 8PZV.pdb
extended conformation
darobactin
D
BamA
darobactin
BamD,E
BamB
BamC
SurA
BamB
BamA
SurA
BAM swing-out
SurA swing-out
BAM 8PZU.pdb
SurA 8PZU.pdb
compact conformation
darobactin

Fig. S10. Structural comparison of BAM–SurA holo insertase complexes. Superimposition on SurA of **A** BAM-SurA swing-in with 8PZ2, **B** BAM–SurA swing-out with 8PZ1, **C** BAM–SurA swing-in with darobactin with 8PZV and **D** BAM–SurA swing-out with darobactin with 8PZU. BAM is shown in green, SurA in purple. The superimposed pdb structures (*42*) are shown in blue for BAM and light blue for SurA. Darobactin is depicted in yellow. On the left side for each superimposition, a zoom in of the SurA binding side is shown.

Table S1.

Cryo-EM data collection and refinement statistics of BAM–SurA with and without BamC and BAM–SurA-darobactin.

	BAM–SurA Swing-in (EMDB-52127) (PDB 9HG6)	BAM–SurA Swing-in BamC (EMDB-52128) (PDB 9HG5)	BAM–SurA Swing-out (EMDB-52129) (PDB 9HG7)	BAM–SurA Swing-out BamC (EMDB-52130) (PDB 9HG8)	BAM–SurA-Daro Swing-in (EMDB-52131) (PDB 9HG9)	BAM–SurA-Daro Swing-out (EMDB-52132) (PDB 9HGA)
Data collection and processing						
Magnification	165,000x	165,000x	165,000x	165,000x	165,000x	165,000x
Voltage (kV)	300	300	300	300	300	300
Electron exposure (e–/Å2)	50	50	50	50	50	50
Defocus range (µm)	-0.6 to -2.0	-0.6 to -2.0	-0.6 to -2.0	-0.6 to -2.0	-0.6 to -2.0	-0.6 to -2.0
Pixel size (Å)	0.73	0.73	0.73	0.73	0.73	0.73
Symmetry imposed	C1	C1	C1	C1	C1	C1
Initial particle images (no.)	16,605,467	16,605,467	16,605,467	16,605,467	4,715,415	4,715,415
Final particle images (no.)	1,088,801	475,381	584,508	325,213	220,992	75,794
Map resolution (Å)	2.73	2.82	2.83	2.90	2.88	3.62
FSC threshold	0.143	0.143	0.143	0.143	0.143	0.143
Map resolution range (Å)	1.6–9.1	1.6–29.8	1.7–9.3	1.8–29.9	1.8-30.0	2.2–46.1
Refinement						
Initial model used	*ab initio, 5LJO*	*ab initio, 5LJO*	*ab initio, 5LJO*	*ab initio, 5LJO*	*ab initio, 7NRI*	*ab initio, 7NRI*
Model resolution (Å)	3.1	3.1	3.1	3.2	3.1	4.0
FSC threshold	0.5	0.5	0.5	0.5	0.5	0.5
Model resolution range (Å)	1.6–6.2	1.6–6.8	1.7–6.7	1.8–7.1	1.8–7.3	2.2–8.5
Map sharpening B factor (Å2)	105.4	95.0	98.5	90.8	91.5	91.1
Model composition						
Non-hydrogen atoms	16,230	16,230	15,241	15,241	13,407	14,217
Protein residues	2,085	2,085	1,953	1,953	1,708	1,818
B factors (Å2) Protein	171.8	171.8	174.1	174.1	145.1	186.7
R.m.s. deviations						
Bond lengths (Å)	0.003	0.003	0.004	0.004	0.002	0.002
Bond angles (°)	0.59	0.59	0.65	0.65	0.50	0.49
Validation						
MolProbity score	1.54	1.54	1.65	1.65	1.47	1.46
Clashscore	5.44	5.44	7.19	7.19	5.00	7.04
Poor rotamers (%)	1.21	1.21	1.16	1.16	0.49	0.00
Ramachandran plot						
Favored (%)	96.9	96.9	96.7	96.7	96.7	97.7
Allowed (%)	3.0	3.0	3.1	3.1	3.3	2.3
Disallowed (%)	0.1	0.1	0.2	0.2	0.0	0.0

Movie S1.
Swing-motion of the BAM–SurA holo insertase complex. The BAM complex is shown in green for BamA and BamB-E are shown as grey transparent surface. The C-terminal BamC domain is not shown. SurA is shown in purple with the P1 domain highlighted in pink. The movie shows a morph starting with the front-view of the swing-in state to the swing-out state and back to swing-in, followed up by a 90° in the z-plane turned view of the process.
